# Cellular and population strategies underpinning neurotoxin production and sporulation in *Clostridium botulinum* type E cultures

**DOI:** 10.1128/mbio.01866-23

**Published:** 2023-11-16

**Authors:** Anna Mertaoja, Gerald Mascher, Maria B. Nowakowska, Hannu Korkeala, Adriano O. Henriques, Miia Lindstrom

**Affiliations:** 1 Department of Food Hygiene and Environmental Health, Faculty of Veterinary Medicine, University of Helsinki, Helsinki, Finland; 2 Instituto de Tecnologia Química e Biológica, Universidade Nova de Lisboa, Lisbon, Portugal; The University of Edinburgh, Edinburgh, United Kingdom

**Keywords:** *Clostridium botulinum*, botulinum neurotoxin, sporulation, phenotypic variation

## Abstract

Toxin production and sporulation are key determinants of pathogenesis in *Clostridia. Clostridium botulinum* produces the most potent toxin known, the botulinum neurotoxin (BoNT), which blocks neurotransmission and causes a life-threatening paralysis called botulism. BoNT production and sporulation share a common regulator Spo0A, which suggests coordination of the two traits. Describing the relationship between toxin production and sporulation is fundamental toward understanding the evolutionary and mechanistic logic and further control of clostridial pathogenesis. Here, we provide the first single-cell resolution analysis of BoNT production and sporulation in *C. botulinum* type E cultures by using a fluorescent reporter to follow the activation of the BoNT gene promoter. BoNT was expressed by a subpopulation of cells and was released through Spo0A-mediated autolysis of vegetative cells or upon release of mature spores. All possible combinations of toxin production and sporulation resided in wild-type *C. botulinum* type E cultures, indicating neither tight co-regulation nor strict independence of the two traits. The population structure and the degree of overall heterogeneity were affected by growth phase and environmental conditions, with cold temperature inducing large diversity and cultural stability, in line with adaptation to fluctuating temperatures that *C. botulinum* type E strains likely encounter in nature. We also observed Spo0A-independent BoNT production by a small cell subpopulation of the *spo0A*-null strain. Our observation of toxin gene activation in the forespore invites speculation on possible alternative biological roles for toxin production by vegetative and sporulating cells and reflection on the evolutionary rationale of toxin production with respect to the ecology of spore-forming pathogens.

**IMPORTANCE:**

Toxin production and sporulation are key determinants of pathogenesis in *Clostridia*. Toxins cause the clinical manifestation of clostridial diseases, including diarrhea and colitis, tissue damage, and systemic effects on the nervous system. Spores ensure long-term survival and persistence in the environment, act as infectious agents, and initiate the host tissue colonization leading to infection. Understanding the interplay between toxin production and sporulation and their coordination in bacterial cells and cultures provides novel intervention points for controlling the public health and food safety risks caused by clostridial diseases. We demonstrate environmentally driven cellular heterogeneity in botulinum neurotoxin and spore production in *Clostridium botulinum* type E populations and discuss the biological rationale of toxin and spore production in the pathogenicity and ecology of *C. botulinum*. The results invite to reassess the epidemiology of botulism and may have important implications in the risk assessment and risk management strategies in food processing and human and animal health.

## INTRODUCTION

Clostridial toxins cause severe diseases in humans and animals, ranging from necrotic enteritis to gas gangrene and tetraplegia. While toxin production probably offers these bacteria a selective advantage in competitive or hostile settings, it is likely to represent a fitness cost. Bacterial populations therefore adjust their toxin production according to prevailing environmental conditions and metabolic states (1–3). Coordinated distribution of traits among individual cells or cell subpopulations would help the population to optimize its energy expenditure and balance between gross consumption and production and growth (4). It is poorly understood how toxin production is coordinated within bacterial populations, and reports on phenotypic heterogeneity in bacterial toxin production (3, 5, 6) or its evolutionary rationales are scarce. Understanding the population-level and cellular strategies bacteria use to coordinate toxin production in response to their environment provides a key to control pathogenesis and prevent related diseases.

The most potent natural toxins with lethal dose at nanogram levels, the botulinum neurotoxins (BoNTs), block neurotransmission in cholinergic synapses (7), causing flaccid paralysis called botulism that may lead to death upon respiratory collapse. BoNTs are mainly produced by growing cultures of *Clostridium botulinum*, which consists of four physiologically, phylogenetically, and metabolically distinct species (Groups I–IV) of anaerobic spore-forming bacteria (8, 9). *C. botulinum* spores are ubiquitous in nature and tolerate starvation, oxygen, and environmental stress, ensuring long-term survival in environments that do not support growth and dissemination of the organism. The spores may contaminate foods and lead to an intoxication known as classical botulism or, analogous to infections caused by *Clostridioides difficile* or *Clostridium tetani*, colonize the gut or deep wounds, respectively, leading to toxicoinfectious botulism. Toxicoinfectious botulism is predominantly caused by the mesophilic Group I strains that are terrestrial of origin and produce types A, B, and/or F toxins and proliferate in the mammalian body temperature, whereas foodborne botulism can be caused by both *C. botulinum* Groups I and II. Psychrotrophic Group II strains produce B, E, or F toxin and are of concern in chilled food production. Group II type E strains prevail in aquatic niches in temperate regions and are found in sediments and in fish (10–13).

In *C. botulinum* cultures, BoNT production peaks at the transition from exponential to stationary growth phase. Population-level studies have shown that sporulation and toxin production are induced in the same time frame (14–17), which suggests common regulation. Indeed, we showed that Spo0A, the master switch of sporulation (18), positively regulates BoNT/E production, most likely by directly binding to the *bontE* promoter (19). The simplest hypotheses arising from these prerequisites assume that toxin production and sporulation are either coupled and concurrent or reciprocally activated Spo0A-driven cellular programs. While the net amounts of toxin and spores at the population level might not differ between the two scenarios, the energy metabolism or evolutionary states of cells would be substantially different. Reciprocal programming of toxin production and sporulation would advocate the role of toxin production in support of vegetative life, while concurrent induction of both traits would assume toxinogenesis in support of longevity through sporulation and, possibly, a role for toxin in the spores. Published studies have solely relied upon population-level net analysis of spores and toxin and have not addressed this relationship at the single-cell level. An asporogeneous “hyper-toxic” *C. botulinum* strain (14) supports reciprocal programming whereas reports showing both traits in wild-type (WT) cultures (20) support concurrent programming. Alternatively, stochastic switching may contribute to the overall fitness of populations under fluctuating conditions (21–23). Understanding the logic of this programming in cultures and cells is fundamental to clarify the relationship and roles of BoNT production and sporulation.

Here, we provide single-cell resolution insight into BoNT production and sporulation in *C. botulinum* cultures. Using a *C. botulinum* Group II type E model system, we demonstrate major differences in population structure and BoNT production in different environmental settings and growth phases. We show that individual cells in a population have distinct roles concerning BoNT production and sporulation and propose alternative biological roles for toxin production by vegetative and sporulating cells. The results introduce a fundamentally new level of understanding of BoNT production and sporulation and have intriguing implications for the biology of spore-forming pathogens.

## MATERIALS AND METHODS

### Experimental concept

We explored BoNT gene (*bontE*) expression and sporulation on single-cell and population levels and studied the effect of growth temperature on the population structure concerning the two traits. First, we validated the SNAP^Cd^ fluorescent reporter system (24) for monitoring the *bontE* promoter (P*bontE*) activity in *C. botulinum* Group II model strain Beluga E1, further referred to as Beluga (supplemental results, Fig. S1). Fluorescence scans and Western blots were used to verify SNAP^Cd^ production and labeling by TMR-Star substrate (Fig. S1). P*bontE* activity was then followed at the single-cell level at different growth phases using fluorescence microscopy in a routine tryptone-peptone-glucose-yeast extract (TPGY) medium (25) and validated against population-level BoNT production using a commercial enzyme-linked immunosorbent assay (ELISA) (supplemental materials and methods, supplemental results, and Fig. S2 and S3).

Next, we studied the effect of growth temperature on population structure concerning BoNT production and sporulation in a medium supporting sporulation and BoNT production (cooked meat medium-TPGY [CMM-TPGY] [26]). Beluga was grown in CMM-TPGY at its routine laboratory growth temperature of 30°C for 96 h or at 10°C for 60 days. SNAP^Cd^ production as a result of P*bontE* activation was monitored by fluorescence microscopy. Membrane dye Mitotracker Green (MTG) and fluorescence and phase-contrast microscopy allowed for detection of different stages of sporulation. Growth phase of the culture was followed by optical density measurements and population-level BoNT concentration was measured with ELISA.

Last, we investigated the role of Spo0A, the master regulator of sporulation (27) and a positive regulator of BoNT production (19), in *bontE* expression in cells by monitoring P*bontE* activation and BoNT and spore production in a Beluga *spo0A* deletion mutant and in a complemented strain at 30°C in CMM-TPGY medium as described above.

All experiments were performed in duplicate or triplicate. Results are presented for a representative replicate culture (range between replicates is reported in parentheses when appropriate, data shown in Fig. S4, S6, and S7).

### Bacterial strains, media, and growth conditions

The bacterial strains and plasmids used are listed in Table 1. Beluga was cultured in TPGY or CMM-TPGY (26) at 30°C in an anaerobic workstation (Don Whitley Scientific Ltd., Shipley, United Kingdom) with an atmosphere of 85% N_2_, 10% CO_2_, and 5% H_2_ or at 10°C in an incubator in airtight jars with AnaeroGen Sachets (Oxoid Microbiology Products, Basingstoke, UK). When appropriate, the media were supplemented with thiamphenicol (15 µg/mL), D-cycloserine (250 µg/mL), and erythromycin (5 µg/mL) (Sigma-Aldrich, St. Louis, MO). *Escherichia coli* strains were grown aerobically at 37°C in Luria-Bertani (LB) broth or on LB agar plates. Chloramphenicol (25 µg/mL) and kanamycin (30 µg/mL) (Sigma-Aldrich) were used for selection.

**TABLE 1:** Bacterial strains and plasmids used

Strain or plasmid	Description	Source or reference
*C. botulinum* E1 Beluga	WT strain	DFHEH/Lindroth^*a*^
Beluga pFT47	WT with pFT47, negative control strain for fluorescence microscopy	This study
Beluga pFT47-P*bontE*	WT with pFT47-P*bontE*, strain for observing toxin promoter activity by fluorescence microscopy	This study
Beluga Δ*spo0A*::bm	*spo0A* deletion mutant	Mertaoja et al. (26)
Beluga Δ*spo0A*::bm pFT47	Δ*spo0A* with pFT47, negative control for fluorescence microscopy	This study
Beluga Δ*spo0A*::bm pFT47-P*bontE*	Δ*spo0A* with pFT47-P*bontE*, strain for observing toxin promoter activity by fluorescence microscopy	This study
Beluga Δ*spo0A*::bm::*spo0A*-wm	Beluga Δ*spo0A* with *spo0A* restored at the original locus	Mertaoja et al. (26)
Beluga Δ*spo0A*::bm::*spo0A*-wm pFT47	Δ*spo0A::spo0A* with pFT47, negative control for fluorescence microscopy	This study
Beluga Δ*spo0A*::bm::*spo0A*-wm *pFT47-*P*bontE*	Δ*spo0A::spo0A* with pFT47-P*bontE*, strain for observing toxin promoter activity by fluorescence microscopy	This study
*E. coli* NEB 5-alpha	Competent strain for cloning	New England Biolabs
*E. coli* CA434	Conjugation donor	Purdy et al. (28)
pFT47	pMTL84121-*SNAP^Cd^*	Pereira et al. and Heap et al. (24, 29)
pFT47-P*bontE*	pFT47 containing promoter region of Beluga toxin gene cluster controlling *SNAP^Cd^*	This study

^*a*^ Strain was obtained from Seppo Lindroth (National Veterinary Institute, Finland/University of California, Davis).

### Reporter strains for single-cell studies

**Fig 1:**
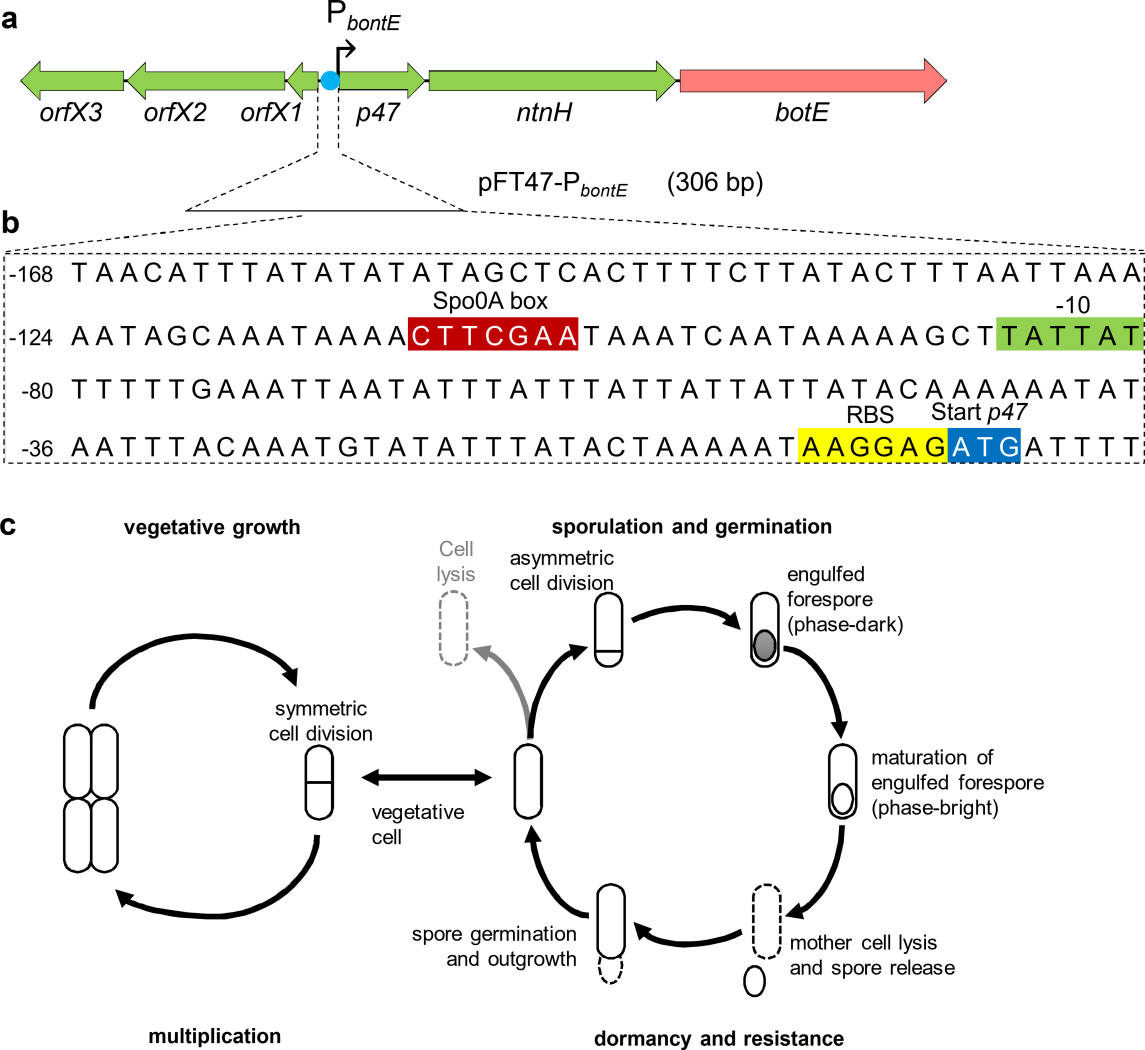
Toxin gene cluster and life cycle of *C. botulinum* E1 Beluga. (a) Genetic organization of the *C. botulinum* E1 Beluga neurotoxin gene cluster. The line below the map shows the extent of the DNA fragment from the *orfX1* and *p47* intergenic region containing the putative toxin gene promoter cloned in pFT47-P*bontE*. (b) The panel shows part of the sequence in pFT47-P*bontE*. The −10 region of a putative σ^A^-type promoter is shown in green. Note that although a canonical −35 region cannot be found, a Spo0A-binding box is present and highlighted in red. The ribosome-binding site and start codon of *p47* are also highlighted. (c) The vegetative (left) and sporulation (right) cycles of *C. botulinum*. Sporulation is induced upon entry into the stationary phase of growth, and cells go through a series of morphological transitions (indicated) that culminate with the formation of highly resilient spores. The spores are released into the environment upon lysis of the mother cell.

To construct plasmid pFT47-P*bontE*, the intergenic region between *orfX1* and *p47* (16) (Fig. 1a and b), containing the putative promoter of the neurotoxin gene cluster (P*bontE*) of Beluga, was PCR-amplified using primers P*bontE*_Fw_BamHI and P*bontE*_R_XhoI (Table 2). The 306-bp PCR product was inserted between the BamHI and XhoI sites of pFT47 (24). Plasmids pFT47 and pFT47-P*bontE* were transformed into *E. coli* CA434 and then conjugated into Beluga WT and into a congenic Δ*spo0A* mutant [Mertaoja et al. (26), Table 1]. The presence of the plasmids was verified by PCR, and the P*bontE-SNAP^Cd^* insert was sequenced.

**Fig 2:**
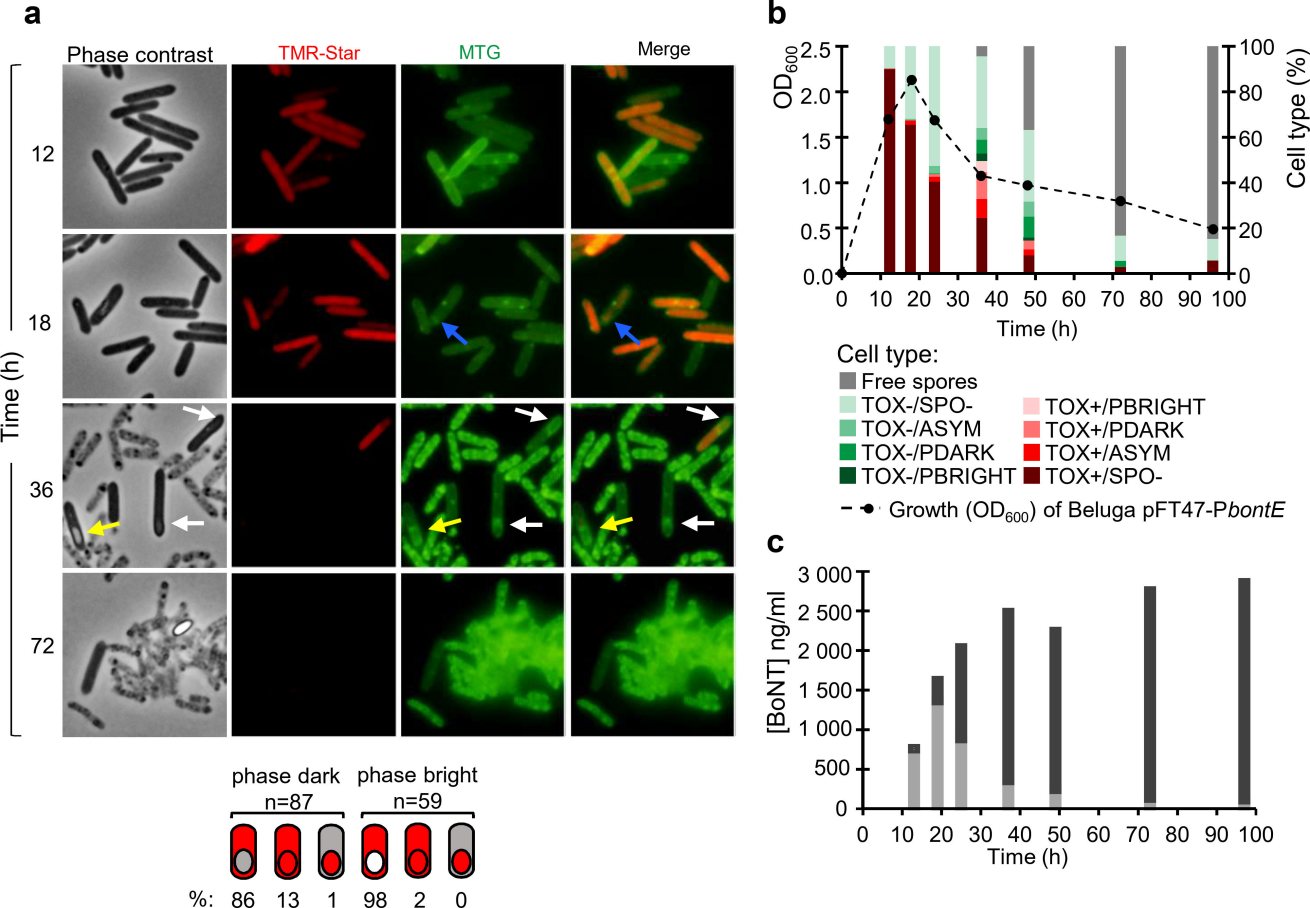
Growth, P*bontE-SNAP^Cd^* expression, sporulation, and BoNT production in CMM-TPGY at 30°C. (a) Samples were collected and labeled with the TMR-Star substrate at the indicated times after inoculation, to monitor expression of the P*bontE-SNAP^Cd^* fusion. The membrane dye MTG was used to visualize the cell and spore membranes. The samples were examined by phase-contrast and fluorescence microscopy to detect dividing or sporulating cells and cells producing the SNAP^Cd^ as an indicator of toxin gene promoter activation (TOX^+^). The merged images show the overlap between TMR-Star (red) and MTG (green, cell membranes and stage of sporulation) channels. The lighter gray cells with irregular outline visible in phase-contrast images of 36- and 72-h samples were considered non-viable and excluded from analysis. In the bottom panel, the location of the TMR-Star-labeled SNAP signal (red) was recorded for sporulating cells with recently engulfed (phase-dark) and matured (phase-bright) forespores (see Fig. 1c for the sporulation cycle). In cells with phase-dark forespores, the SNAP signal was found in the mother cell, or in both mother cell and forespores, or confined to the forespore. In sporulating cells with phase-bright forespores, the signal was found in the mother cell compartment only, except for one cell with signal in both the mother cell and the forespore. The figure combines results from all three replicates. Scale bar: 5 µm. (b) Population structure at each sampling time point concerning toxin production (TOX^+^ or TOX^−^) and sporulation [SPO^−^, vegetative cell; ASYM, asymmetrically dividing cell (blue arrow); PDARK, phase-dark forespore (white arrows); PBRIGHT, phase-bright forespore (yellow arrows)]. A total number of cells scored for each time point were at 12 h, *n* = 954; 18 h, *n* = 1,612; 24 h, *n* = 364; 36 h, *n* = 406; 48 h, *n* = 76; 72 h, *n* = 36; and 96 h, *n* = 56. Growth was followed by measuring optical density at 600 nm. (c) The neurotoxin concentration in cell sediments (light gray bars) and culture supernatants (dark gray bars) was determined by ELISA. Data shown are from one representative experiment out of three independent experiments. See Fig. S4 for other replicates.

**TABLE 2:** PCR primers used

Primer	Sequence (5′→3′)*^a^*	Use
P*bontE*_Fw_BamHI	aattGGATCCcccttcccttctcaatagtt	Amplification of the *bontE* promoter region
P*bontE*_R_XhoI	aattCTCGAGataaaatcatctccttatttttagtat	Amplification of the *bontE* promoter region

^*a*^ Engineered restriction sites are shown in uppercase letters.

### Neurotoxin ELISA

BoNT concentration was determined from 1-mL cell culture samples using commercial ELISA Antigen Capture to Detect Botulinum Toxin E kits (Tetracore, Rockville, MD) as described earlier (19).

### Microscopy and image analysis

For SNAP^Cd^ detection, 200- to 600-µL samples were collected from cell cultures at the desired time points and labeled with 500 nM of the TMR-Star substrate (New England Biolabs) for 30 min. The cells were collected by centrifugation (6,000 × *g*, 3 min, room temperature [RT]), washed twice with 1 mL of PBS, resuspended in 200 µL of PBS containing 0.5 µg/mL of the membrane dye MTG (Molecular Probes, Invitrogen, Carlsbad, CA), incubated in the dark for 3 min, collected by centrifugation, and washed with PBS. Finally, 200 µL of a fixative (0.025% glutaraldehyde and 0.04% formaldehyde) was added, and the cells were incubated for 1 h at RT, protected from light. The fixed cells were washed with PBS and resuspended in GTE buffer (50 mM glucose, 20 mM Tris, pH 8.0, and 10 mM EDTA). The fixed samples were protected from light and stored at 4°C for no longer than 24 h.

For microscopy, the cells were mounted on glass slides coated with 1.7% agarose and observed with a Leica DM4000B microscope equipped with a Leica HCX PL FLUOTAR 100× objective and Leica TX2 and I3 filter cubes to detect the signals from SNAP^Cd^-TMR-Star and MTG, respectively. Images were collected with an Olympus DP70 camera using the Cell^P software (Olympus Life Science, Waltham, MA). Exposure times of 20, 200, and 50 ms were used for phase contrast or the TX2 and I3 filters, respectively. Background fluorescence was determined from negative control images (Beluga pFT47 labeled with TMR-Star) or from non-viable cells in sample images (i.e., light gray cells with irregular shape showing in phase-contrast images). The image brightness was normalized by subtracting the background signal from the fluorescence intensity in cells in sample images. Thus, any cells in sample images showing fluorescence after normalization were considered positive regarding P*bontE* activity and SNAP^Cd^ production. Percentage of SNAP^Cd^-producing cells in the population was determined with Fiji (30, 31) (http://fiji.sc/Fiji): the phase-contrast image was thresholded so that individual cells were selected, cell outlines were transferred onto the respective TMR-Star image, and average signal intensity within each outline was calculated. MTG staining was used to determine sporulation status (vegetative or sporulating; for sporulating cells the stage of sporulation) for each analyzed cell. Non-intact or non-viable (lighter gray with irregular shape in phase-contrast images) cells, overlapping cells, or cells that were out of focus were excluded from the analysis.

For characterization of twin sporulation, 1 mL of cell culture was collected by centrifugation as described above, washed with 1-mL PBS, and resuspended in 1-mL PBS containing the membrane dye FM4-64 [*N*-(3-triethylammoniumpropyl)-4-(6-(4-(diethylamino)phenyl) hexatrienyl)pyridinium dibromide, final concentration 1 µg/mL] and DNA dye DAPI (4′,6-diamidino-2-phenylindole, dihydrochloride, final concentration 5 µg/mL) (Molecular Probes, Invitrogen). The samples were incubated in the dark for 4 min and washed with PBS. The samples were observed using the Leica DMi8 inverted fluorescent microscope equipped with HC PL APO 100×/1.40 OIL PH3 objective, a Leica filter cube DA/FI/TX, and a custom-made filter from Chroma to visualize FM4-64. Images were captured with the Hamamatsu Orca Flash V2 LT camera using the Metamorph Basic Acquisition for Microscope software.

For live/dead analysis of cells, 1 mL of culture was collected and washed with 1-mL PBS. The cell sediment was resuspended in 500-µL PBS, and 1.5 µL of LIVE/DEAD BacLight reagent containing 1.67-mM Syto9 and 10-mM propidium iodide was added (Molecular Probes, Invitrogen). The samples were incubated in the dark for 15 min, washed once with PBS, mounted on agarose-coated slides as above, and imaged with the Leica DMi8 inverted microscope as described above.

### Statistical analysis

Correlation between intracellular BoNT concentration and the percentage of TOX^+^ cells at different time points was tested with Pearson correlation (two-tailed). Pearson’s *χ*^2^ test of independence was used to determine the association between sporulation and neurotoxin production. Statistical analyses were performed using SPSS Statistic version 25 (IBM, Armonk, NY).

## RESULTS

### Phenotypic heterogeneity in P*bontE* expression within a population

To study the relationship between BoNT production and sporulation, Beluga pFT47-P*bontE* cultures were established in CMM-TPGY at 30°C. Samples for fluorescence and phase-contrast microscopy, BoNT, and spore measurements and for growth monitoring were taken at the late-logarithmic (12 h), transition (18 h), early stationary (24 h), and stationary (36, 48, 72 and 96 h after inoculation) phases of growth.

Transcription from the neurotoxin gene promoter (TOX^+^ cells) was detected during both vegetative growth (SPO^−^ cells) and sporulation (SPO^+^) (Fig. 1c and 2a). A non-toxic non-sporulating (TOX^−^/SPO^−^) subpopulation was nevertheless present at all sampling times. In the late-logarithmic and transition phases of growth, the proportion of TOX^+^ cells in the population reached 90% (Fig. 2b [84%–93%; Fig. S4]) and 67% (Fig. 2b [75%–93%, Fig. S4]), respectively, followed by a decline to 44% (Fig. 2b [49%–71%; Fig. S4]) at the onset of stationary phase. A strong positive correlation (Pearson’s *r* = 0.69, *P* = 0.001) was detected between the percentage of TOX^+^ cells and the intracellular concentration of BoNT (Fig. 2b and c).

Cells with asymmetric division septa (Fig. 2a, blue arrow), indicating commitment to sporulation (Fig. 1c), were detected in the late-logarithmic cultures and onward. Cells with engulfed forespores, however, indicating late stages of sporulation (Fig. 1c), were not visible until early stationary phase when 1.1% (Fig. 2b [0.2%–0.4%; Fig. S4]) of the cell population consisted of mother cells with engulfed phase-dark (Fig. 2a, white arrows) or phase-bright forespores (Fig. 2a, yellow arrows; Fig. 1c). Expression of P*bontE-SNAP^Cd^* was detected both in asymmetrically dividing cells and in cells with engulfed forespores. Importantly, expression of P*bontE-SNAP^Cd^* was generally confined to the mother cell, suggesting that transcription from the neurotoxin promoter is largely a mother-cell property in the tested conditions (Fig. 2a, bottom). Only 13% of TOX^+^ sporulating cells with phase-dark forespores (*n* = 87) showed a signal in both the mother cell and forespore, with this proportion declining to 2% at later stages of sporulation in cells with phase-bright forespores (*n* = 59, Fig. 2a, bottom). Population heterogeneity was greatest at 36 h (Fig. 2b): all combinations of toxin expression and sporulation in cells (TOX^+^/SPO^+^, TOX^+^/SPO^−^, TOX^−^/SPO^+^, and TOX^−^/SPO^−^ cells), as well as free spores, were detected.

The late-stationary phase (48‒96 h) was dominated by free spores and cell debris due to cell lysis (Fig. 2a, 72 h). Interestingly, a stable TOX^+^ subpopulation of vegetative cells (20%‒32%, Fig. 2b [15%‒38%; Fig. S4]) persisted despite the declining culture density. As none of the late TOX^+^ cells were sporulating at the late hours, it is possible that these cells represent outgrowth from germinated spores.

To determine the role of the inoculum as the source of differently behaving cell subpopulations, we inoculated CMM-TPGY batches with a single colony of Beluga pFT47-P*bontE* from an agar plate. These cultures also showed heterogeneity in BoNT gene promoter activity and sporulation (supplemental results, Fig. S5); thus, heterogeneity appears to be inherent in *C. botulinum* populations.

Together, these results show that toxin expression occurs both during vegetative growth and during sporulation and is, in both cases, heterogeneous, as both TOX^+^ and TOX^−^ cells were detected. The implication is that not all the cells contribute to the net level of BoNT detected in cultures. During sporulation, toxin gene promoter activity appeared to localize mainly in the mother cells.

### Low temperature supports long-lived and diverse populations

**Fig 3:**
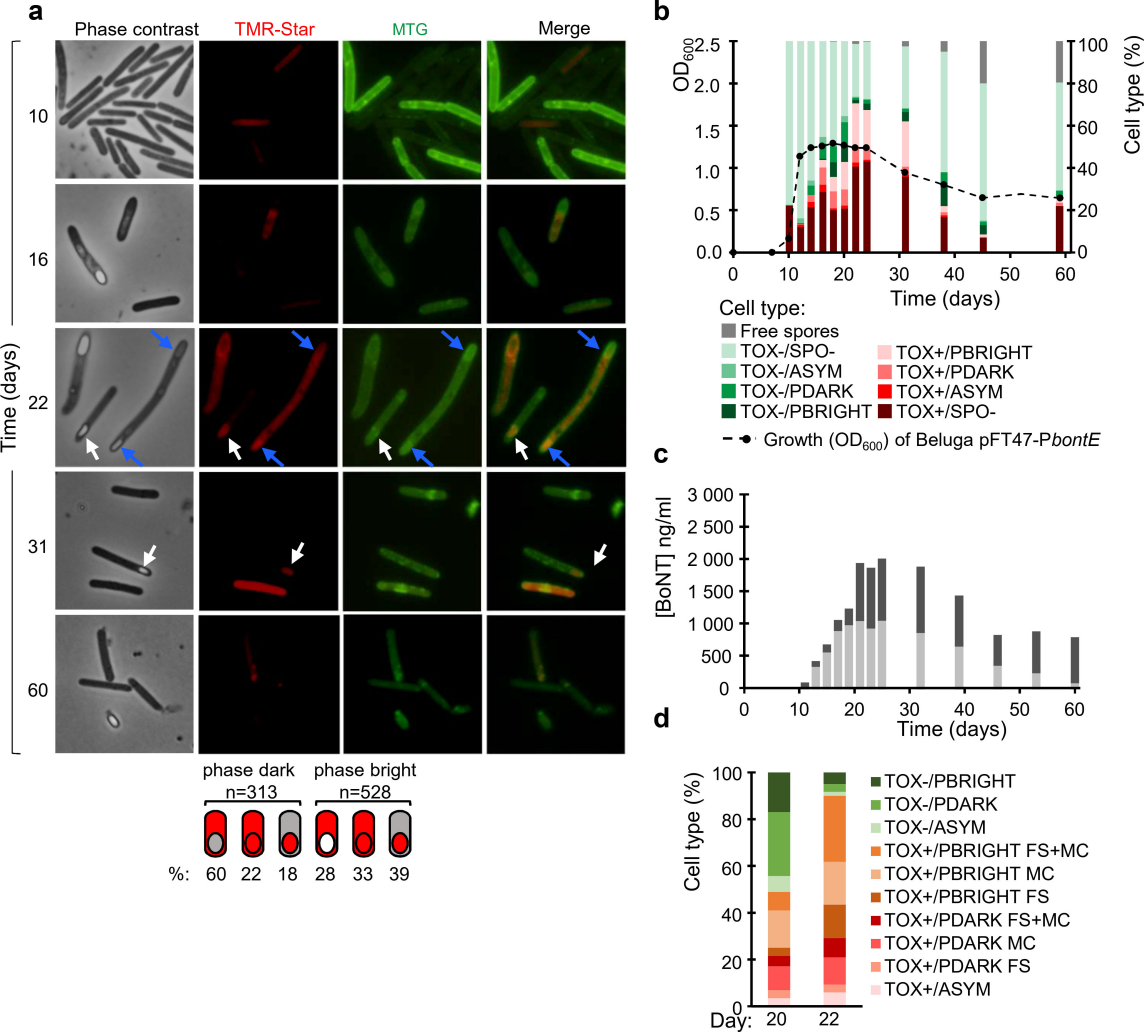
Growth, P*bontE-SNAP^Cd^* expression, sporulation, and BoNT production in CMM-TPGY at 10°C. (a) Samples were collected and labeled with the TMR-Star substrate at the indicated times after inoculation, to monitor expression of the P*bontE-SNAP^Cd^* fusion. The membrane dye MTG was used to visualize the cell and spore membranes. The samples were examined by phase-contrast and fluorescence microscopy to detect dividing or sporulating cells and cells producing the SNAP^Cd^ as an indicator of toxin gene promoter activation (TOX^+^). The merged images show the overlap between TMR-Star (red, TOX^+^ cells) and MTG (green, cell membranes and stage of sporulation) channels. Scale bars: 5 µm. The bottom panel shows the localization of the SNAP signal (red) for sporulating cells at different stages of sporulation (see Fig. 1c for the sporulation cycle). The SNAP signal was found in the mother cell, or in both mother cell and forespores, or confined to the forespore (white arrows). Blue arrows indicate a cell with engulfed forespores at both poles. (b) Population structure at each sampling time with respect to P*bontE-SNAP^Cd^* expression (TOX^+^ or TOX^−^ cells) and sporulation (SPO^−^, vegetative cells; stages of sporulation [SPO^+^, see Fig. 1c for the sporulation cycle]; ASYM, asymmetrically dividing cell; PDARK, phase-dark forespore; and PBRIGHT, phase-bright forespore). A total number of cells scored for each time point were at day 10, *n* = 408; day 12, *n* = 978; day 14, *n* = 693; day 16, *n* = 535; day 18, *n* = 552; day 20, *n* = 208; day 22, *n* = 372; day 24, *n* = 260; day 31, *n* = 271; day 38, *n* = 137; day 45, *n* = 200; and day 60, *n* = 137. Growth was followed by measuring optical density at 600 nm. (c) The neurotoxin concentration in cell sediments (light gray bars) and culture supernatants (dark gray bars) was determined by ELISA. Data shown are from one representative experiment out of two independent experiments. See Fig. S6 for the other replicate. (d) Sporulating (SPO^+^) cell population at day 20 (*n* = 88) and day 22 (*n* = 120) of growth shows that in a subpopulation of cells, sporulation is initiated before P*bontE-SNAP^Cd^* expression. FS, forespore-specific SNAP signal (Fig. 3a, white arrows); MC, mother-cell-specific SNAP signal; FS^+^ MC, SNAP signal detected in both mother cell and forespore. In all asymmetrically dividing TOX^+^ cells, SNAP was detected in both mother cell and forespore.

BoNT production has been reported at environmental temperatures down to 3°C (32). Differential neurotoxin gene expression patterns at low and high temperatures suggest temperature-related regulation of neurotoxin synthesis (16, 33). Therefore, we studied the effect of low temperature on P*bontE* expression and sporulation in Beluga pFT47-P*bontE* grown in sporulation medium (CMM-TPGY) at 10°C. Samples were taken between 10 and 60 days after inoculation to measure growth, sporulation, and neurotoxin levels (Fig. 3a and b; Fig. S6).

Similar to the population grown at 30°C, the population at 10°C contained all possible combinations of TOX and SPO states in cells (Fig. 3a and b; Fig. S6). However, unlike at 30°C, where TOX and SPO showed distinct temporal patterns and overlapped only during a short time window during the transition phase (Fig. 2b), at 10°C, all the various cell types were observed throughout the 60-day experiment (Fig. 3b; Fig. S6). TOX^+^ cells appeared on day 10, and more than half of the cells scored were TOX^+^ after 1 month. Even after 2 months, the population was dominated by vegetative cells (Fig. 3a, 60 days), whereas, at 30°C, the 4-day-old late-stationary population mainly consisted of free spores and lysed cells. The intracellular neurotoxin levels measured at 10°C peaked between days 20 and 31 and declined gradually during the last 4 weeks of the study, suggesting the presence of a stable and active TOX^+^ population in the cold (Fig. 3c). The decline in total toxin concentration at the late stages of the 2-month long experiment is likely due to degradation of protein toxin and the low level of new toxin synthesis.

### Low temperature activates neurotoxin promoter in the forespore

While, at 30°C, sporulating cells appeared only after the TOX^+^ population and intracellular toxin concentration declined (see above), at 10°C, the SPO^+^ population peaked (44%, day 20) before the peak of the TOX^+^ population (70%, day 22) (Fig. 3b [30%–44% and 68%–70% on days 20/22 and 22/24, respectively; Fig. S6]). Interestingly, a decline in the TOX^−^/SPO^+^ cell population between days 20 and 22 (22%–3%) coincided with an increase in the TOX^+^/SPO^+^ population (22%–30%) (Fig. 3b and d [15%–22% to 3% and 15%–22% to 24%–30%; Fig. S6]). This suggests that, as opposed to the strict temporal regulation of P*bontE* expression and sporulation at 30°C, temporal regulation of the two traits was less stringent at 10°C and enabled a subpopulation of cells where BoNT production is initiated only after the onset of sporulation.

While, at 30°C, the SNAP^Cd^ signal was almost exclusively confined to the mother cell (Fig. 2a, bottom; see also above), at 10°C, the majority (*n* = 503, 60%) of TOX^+^ cells with engulfed forespores (*n* = 841) observed at any sampling time showed the SNAP^Cd^ signal in the forespore (Fig. 3a, bottom, and Fig. 3d). Of 503 sporulating cells scored, 48% (*n* = 241) showed the SNAP^Cd^ signal in both mother cell and forespore, and in 52% (*n* = 262), the signal was confined to the forespore. The SNAP^Cd^ signal was more likely confined to the forespore in cells with phase-bright forespores than in cells containing phase-dark forespores (Fig. 1c and 3a, bottom). The forespore-specific SNAP^Cd^ signal suggests that low temperature acts as a signal that triggers an as-yet unknown regulatory mechanism of BoNT gene transcription in the developing forespore, independent of the regulatory circuits in the mother cell.

### Low temperature co-activates sporulation and toxin production in the population

At 10°C, there was a significant association between sporulation and SNAP^Cd^ production [*χ*²(1) = 750.6, *P* < 0.001]: in the SPO^+^ population (*n* = 1,752, all time points), 54% of the cells were also TOX^+^, whereas, in the SPO^−^ population (*n* = 9,575), only 22% of the cells were TOX^+^. In the TOX^+^ population (*n* = 3,038), 31% of the cells were SPO^+^, but, in the TOX^−^ population (*n* = 8,289), only 10% of the cells were SPO^+^.

At 30°C, only a transient temporal overlap of P*bontE* expression and sporulation was detected during the early stationary growth phase (36 h), where 66% of SPO^+^ cells (*n* = 281) were TOX^+^ and 37% of vegetative cells (SPO^−^, *n* = 471) were TOX^+^. Likewise, in the TOX^+^, subpopulation (*n* = 357), 52% of the cells were also SPO^+^, whereas, in the TOX^−^ population (*n* = 395), only 25% of the cells were SPO^+^. Association between TOX^+^ and SPO^+^ states was significant at this growth phase [*χ*²(1) = 58.7, *P* < 0.001] and insignificant at other time points at 30°C due to sequential activation of the two traits with negligible overlap.

### Low temperature induces twin sporulation

**Fig 4:**
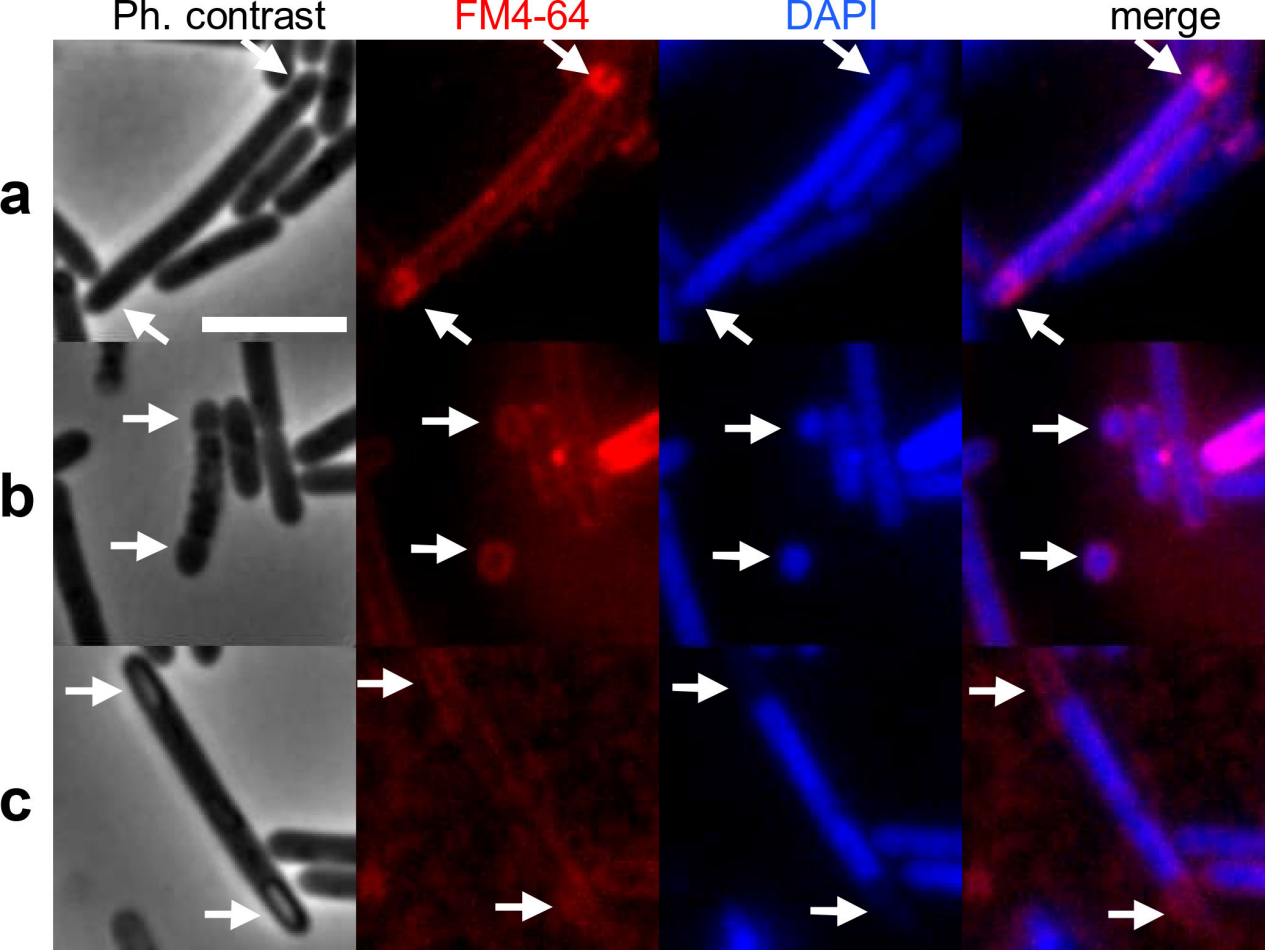
Twin sporulation of Beluga WT pFT47-P*bontE* in CMM-TPGY at 10°C. Cell membranes were labeled with FM4-64 and DNA with DAPI. Merge shows the overlay of FM4-64 and DAPI labeling. White arrows indicate (a) bipolar asymmetric division, (b) abortive disporulation where mother cell lacks DNA and results in cell death, and (c) twin phase-bright forespores indicating successful completion of twin sporulation. Scale bar: 5 µm.

A small but reproducibly observed subpopulation of sporulating cells with asymmetric septa or engulfed forespores in both cell poles was observed at low temperature (Fig. 3a, blue arrows). Bipolar division leads to the production of two spores per cell, a process termed twin sporulation (34–36). DNA and membrane staining (Fig. 4) revealed that bipolar division occurred in 3.3% (*n* = 34, out of 1,042) of the sporulating cells. Twin sporulation, however, requires the presence of a copy of the genome in the mother cell and in each of the two developing spores (34–36). We found that 18% (*n* = 6) of cells dividing at the two poles lacked DNA in either the mother cell (Fig. 4b) or one of the forespores. These cells are reminiscent of the abortive disporic forms described in *Bacillus subtilis* (36) and suggest that the twin sporulation pathway is prone to errors. Out of sporulating cells with phase-bright forespores (*n* = 221), 1.8% (*n* = 4) had phase-bright forespores at both poles (Fig. 4c), demonstrating that bipolar division led to the formation of twin spores. Twin sporulation suggests relaxed control of DNA replication at 10°C, leading to the formation of more than two chromosomes, as well as temporal changes in the sporulation pathway that normally prevent the formation of a second asymmetric septum through the activity of SigE in the mother cell in both *B. subtilis* and several *Clostridia* (36, 37).

### Spo0A-independent P*bontE* activation

**Fig 5:**
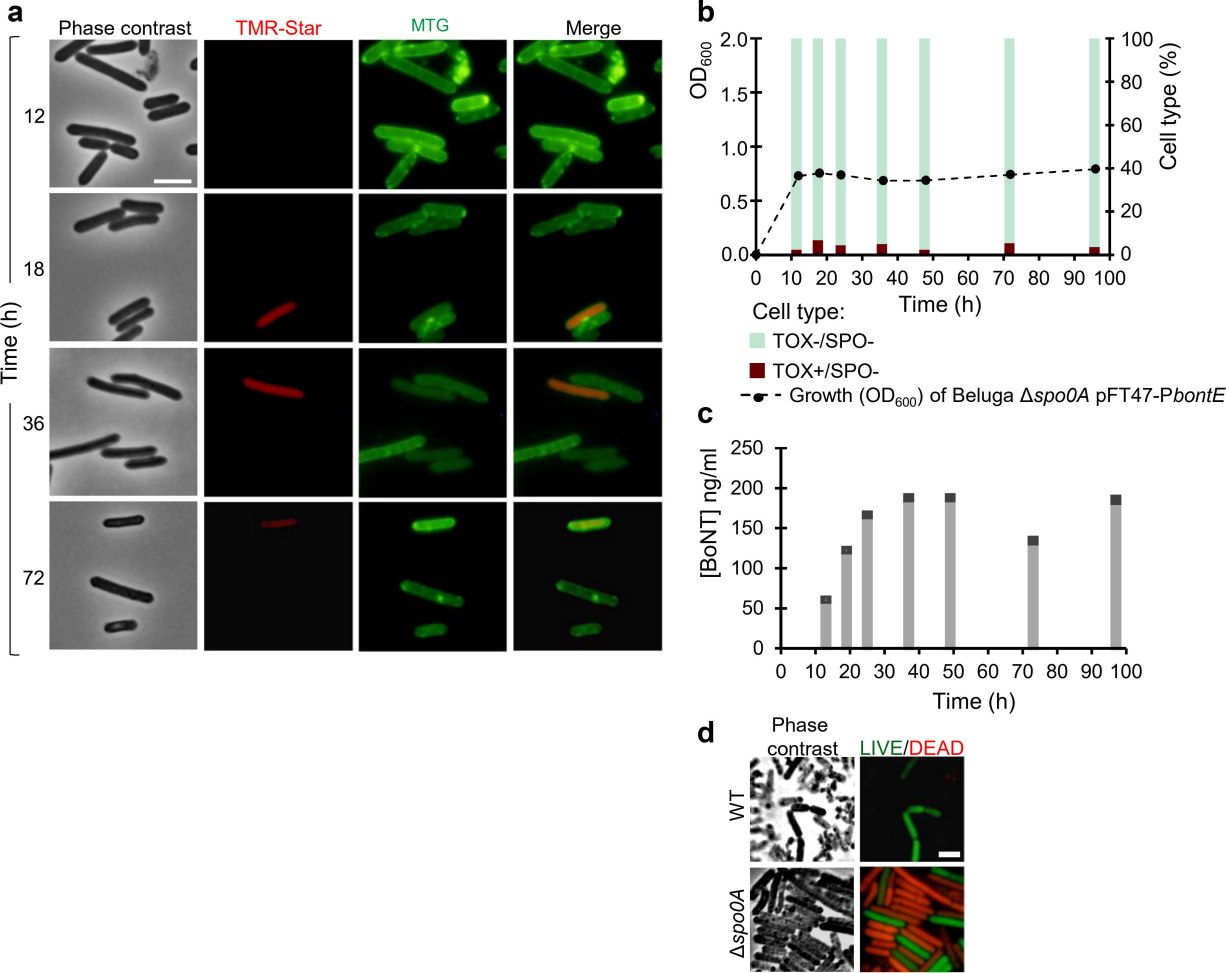
Growth, P*bontE-SNAP^Cd^* expression, sporulation, and BoNT production in a Δ*spo0A* mutant in CMM-TPGY at 30°C. (a) Samples were collected and labeled with the TMR-Star substrate at the indicated times after inoculation, to monitor expression of the P*bontE-SNAP^Cd^* fusion. The membrane dye MTG was used to visualize the cell and spore membranes. The samples were examined by phase-contrast and fluorescence microscopy to detect dividing or sporulating cells and cells producing the SNAP^Cd^ as an indicator of toxin gene promoter activation (TOX^+^). The merged images show the overlap between TMR (red) and MTG (green) channels. (b) Population structure at each sampling time point concerning toxin production (TOX^+^ or TOX^−^) and sporulation. All observed cells were non-sporulating vegetative cells (SPO^−^). A total number of cells scored for each time point were as follows: 12 h, *n* = 736; 18 h, *n* = 657; 24 h, *n* = 2,648; 36 h, *n* = 819; 48 h, *n* = 700; 72 h, *n* = 557; and 96 h, *n* = 337. Growth was followed by measuring optical density at 600 nm. (c) Neurotoxin concentrations in cell pellets (light gray bars) and culture supernatants (dark gray bars) were determined with ELISA. Data shown are from one representative experiment out of three independent replicate experiments. See Fig. S7 for other replicates. (d) LIVE/DEAD analysis of Beluga Δ*spo0A* pFT47-P*bontE* and WT pFT47-P*bontE*. Cells were collected at 48 h after inoculation, stained with a commercial LIVE/DEAD kit, and examined by phase-contrast and fluorescence microscopy. The LIVE/DEAD images were prepared by combining the images of the syto 9 (green, live cells) and propidium iodide (red, dead cells) stained samples. Scale bar, 5 µm.

We have shown that Spo0A is a regulator of not only sporulation but also BoNT production (19). Spo0A binds to a putative Spo0A box in the BoNT gene promoter, and an insertional *spo0A* mutant produced no spores and substantially reduced BoNT levels of down to 10% of those measured in WT cultures (19) (Fig. 1b). The cells in these cultures retained BoNT intracellular for extended periods of time, while WT cells would release BoNT upon vegetative or mother cell lysis (19). To examine the role of Spo0A in P*bontE* expression at the single-cell level, we transferred the pFT47-P*bontE* plasmid to an in-frame *spo0A* deletion mutant (26). As expected, the mutant did not form spores, and its neurotoxin production was significantly diminished but not abolished. Interestingly, instead of a low-level toxin gene activation throughout the cell population, a small subpopulation of TOX^+^ cells (2%‒7%, *n* = 346‒2,422) was detected at every sampling point throughout the 96-h experiment (Fig. 5a and b; Fig. S7). Notably, a TOX^+^ subpopulation of a similar size was repeatedly maintained in a passage of four subsequent cultures. Successful restoration of the WT phenotype by *in trans* complementation with the *spo0A* gene confirmed that the sporulation and toxin production phenotypes were caused by the absence of *spo0A* (Fig. S8). These findings suggest that an as-yet unknown regulatory mechanism ensures neurotoxin production in a subpopulation of cells independently of Spo0A. It is not known how such a Spo0A-independent BoNT regulation manifests in a WT population and whether it affects the WT population structure.

Unlike in WT cultures, where the optical density dropped in stationary phase and up to 97% of the neurotoxin was released to the culture supernatant (Fig. 2b and c), the Δ*spo0A* cultures maintained the optical density at stationary phase, and only 2.5% of the neurotoxin detected was released to the culture supernatant (Fig. 5c). By phase-contrast and fluorescence microscopy, the late-stationary Δ*spo0A* pFT47-P*bontE* cells maintained their shape, as opposed to WT cells that autolyzed or sporulated. To further investigate the fate of the Δ*spo0A* pFT47-P*bontE* cells in the late-stationary phase, we used LIVE/DEAD staining and fluorescence microscopy (Fig. 5d). Interestingly, 75% of the cells with intact shape in phase-contrast microscopy stained DEAD, indicating membrane damage and cell death, whereas in WT cultures, over 90% of the few intact cells stained LIVE. These findings suggest a role for Spo0A in cell lysis, either through sporulation and/or, upon its completion, in mother-cell lysis, or during growth, or both.

## DISCUSSION

Studies of bacterial toxin production and sporulation have relied on batch measurements of toxins and spores in bacterial cultures. Gene expression and trait relationships at the single-cell level have remained unaddressed. Elucidating the relationship between toxin production and sporulation is a prerequisite for understanding the evolutionary rationale and regulation of these traits. In this context, knowledge on the population structure with respect to toxin and spore production is essential and opens novel approaches to control toxinogenesis. Here, we provide the first insight into single-cell and population-level strategies in neurotoxin production and sporulation by *C. botulinum* type E. The results introduce a fundamentally new level of understanding of BoNT production and its relationship to sporulation and have several important implications for the biology and pathogenesis of *C. botulinum* and other spore-forming pathogens.

We show that *C. botulinum* cultures are partitioned into subpopulations with all possible cellular modes of toxin production and sporulation (TOX^+^/SPO^+^, TOX^+^/SPO^−^, TOX^−^/SPO^+^, and TOX^−^/SPO^−^). While heterogeneity appears to be an inherent property of the cultures, the population structure and the degree of overall heterogeneity are substantially affected by external stimuli, including growth phase, environmental conditions such as temperature and growth medium, and at least to some extent by the history of the cells. Further research is warranted to reveal the underlying mechanisms driving the observed heterogeneity. Whether the subpopulations are a result of stochastic gene expression or coordinated division of labor in response to external cues (4, 38) and whether the observed heterogeneity represents a bet-hedging strategy to enhance the probability of the population survival in a fluctuating environment remain to be understood (21, 22, 39, 40).

Sporulating cells release BoNT via mother-cell lysis upon releasing the mature spore, but the mechanism of BoNT release from non-sporulating cells is currently not known. As our study showed that the Δ*spo0A* mutant kept most of its BoNT in the cells that were mainly dead, it seems plausible that non-sporulating cells release BoNT through Spo0A-mediated autolysis. This theory is further supported by the strong correlation between BoNT accumulation in the culture supernatant and the decline in optical density in conditions that do not support sporulation (supplemental results). However, an active secretion mechanism, such as the ones identified in several clostridial species producing large protein toxins (41–45), cannot be ruled out. Our data showing accumulation of BoNT in a log-phase culture supernatant before massive cell lysis or sporulation at the stationary growth phase may support the presence of an alternative, perhaps growth-phase-dependent BoNT release mechanism that does not rely on cell lysis.

Population-level studies in batch cultures show an overlap of peak neurotoxin production and entry into sporulation (14, 16, 46). As the sporulation master switch, Spo0A, also regulates BoNT production (19), the two traits have been assumed to be linked in cells, as has been demonstrated for enterotoxin production by *Clostridium perfringens* and C_2_ toxin production by *C. botulinum* (47, 48). Our findings showing that environmental conditions greatly affect the ratio of toxinogenesis and sporulation in cultures and the presence of all four possible cellular modes indicate that BoNT production and sporulation are neither tightly co-regulated nor strictly independent traits but more likely appear as stochastically activated Spo0A-controlled programs. It remains to be identified how the Spo0A switch works and how the level of its activation contributes to the partitioning of the population into various cell types.

**Fig 6:**
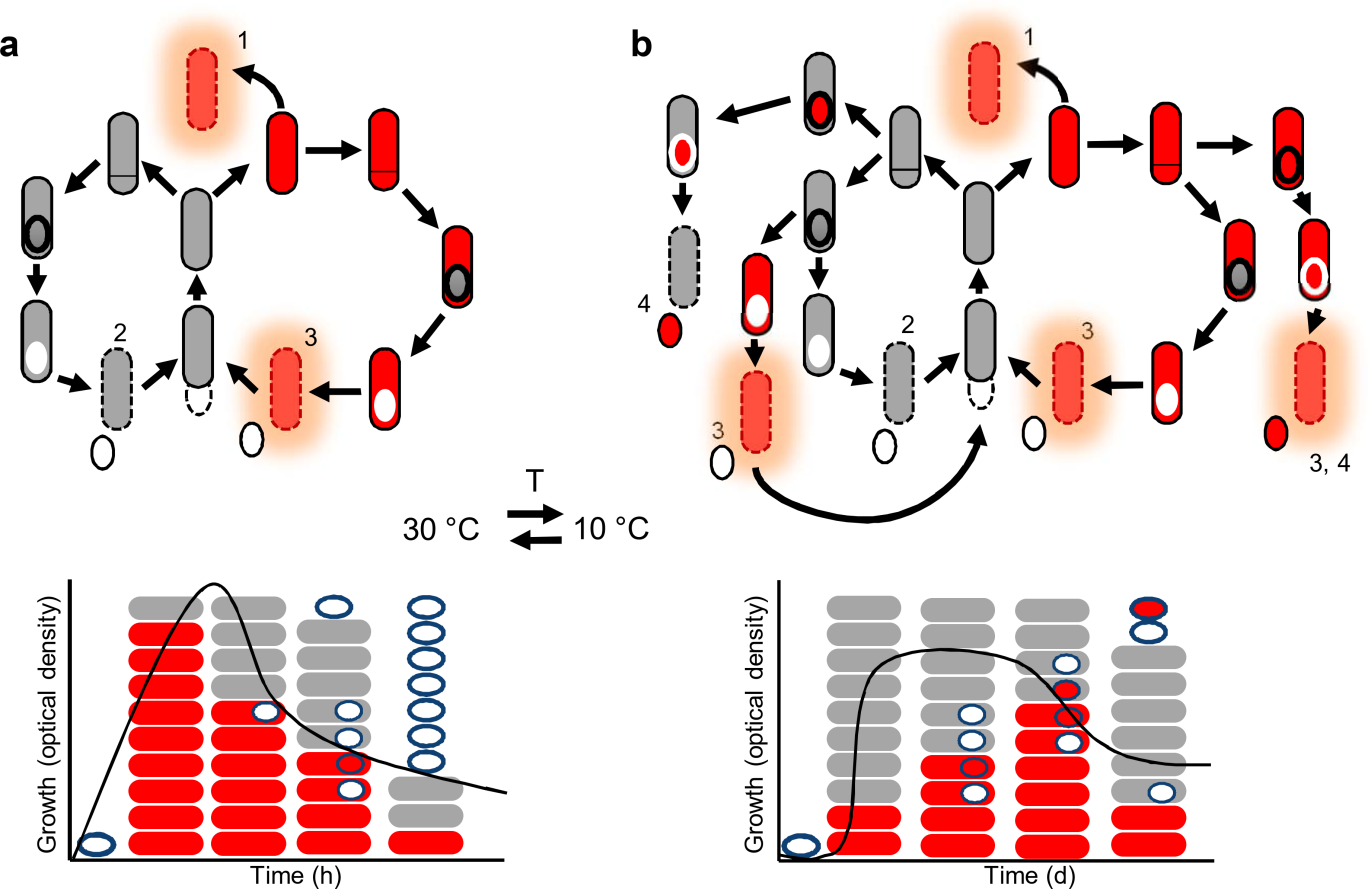
Schematic model of population structure and cellular fates of *C. botulinum* E1 Beluga grown at 30°C and 10°C. Cell fates of differently behaving *C. botulinum* E1 Beluga cells at 30°C (a) and 10°C (b). 1. Autolysis of BoNT-producing vegetative cells maintains a suitable growth environment. 2. Sporulating cells ensure long-term survival. 3. BoNT release upon sporulation helps to maintain growth-supporting conditions and may support immediate re-germination of the spores. 4. BoNT-containing spores may germinate in conditions not optimal for growth, and toxin released upon germination might contribute toward a microenvironment suitable for colonization and growth or eliminate competition. BoNT in spores might also allow germination spatially and temporally distant from the site of sporulation. The two bottom panes represent the population structure at 30°C and 10°C with respect to toxin production and sporulation. Red color indicates toxin synthesis in cells and forespores. Higher temperature (T) supports a fast-growing population with temporal coordination of BoNT production and sporulation. Massive toxin production is followed by synchronized sporulation and rapid culture collapse. Populations at low temperature are characterized by stable and prolonged BoNT production and balanced sporulation and re-germination.

The fast-growing and relatively homogeneous culture observed at 30°C underwent rapid cell death and culture collapse. In contrast, at cold temperature (10°C), the population appeared more heterogeneous and balanced: cell division, BoNT expression, and sporulation did not follow such a strict temporal pattern, and BoNT production and various stages of sporulation overlapped for as long as 6–7 weeks. An active vegetative cell population was present for the entire 2-month follow-up period, and only subtly declining culture density suggested a balance between cell lysis, cell division, sporulation, and germination. The cold environment also allowed partial penetrance of twin sporulation (34–36) (Fig. 4), which may have resulted from a more relaxed control of cellular functions like DNA replication and asymmetric division and may be important for propagation. There was a negligible difference between the maximum concentrations of BoNT measured at 10°C and 30°C. This might suggest that BoNT production is adapted to fluctuating temperatures that *C. botulinum* type E, as our model strain Beluga, likely encounters in its natural environment in aquatic systems of the Northern Hemisphere (10–13). It is probable that the observations at 10°C are more relevant for the biology of Beluga than those at 30°C and that a cold environment is better suited for supporting a versatile and enduring population (Fig. 6) that is likely adaptable to environmental fluctuations and stress (21, 22, 39). Of note, future research will demonstrate how readily the current findings on *C. botulinum* (Group II) type E apply to other *C. botulinum* strains and different biological or epidemiological contexts. *C. botulinum* type E strains are psychrotrophic, non-proteolytic, aquatic, and practically unassociated with toxicoinfectious botulism. On the contrary, *C. botulinum* (Group I) type A represents a different species with different ecological and epidemiological features and causes toxicoinfections of the gastrointestinal tract and wounds. Moreover, both physiological groups (I and II) possess unique properties in the genetics and regulation of BoNT production. Therefore, the present data should not be translated to the ecology or epidemiology of *C. botulinum* (Group I) type A strains. Similar experimentation on a type A strain, preferably growing in a gut infection model, will likely open up completely novel horizons on the epidemiology of botulism.

*C. botulinum* type E spores predominate in aquatic sediments and in the gastrointestinal tract of fish and sea mammals. Vegetative growth and subsequent BoNT production probably occur in decaying organic matter of animal or plant origin, which provides anaerobic and nutritious conditions for spore germination and BoNT production. Scavenging fish or toxin-immune invertebrates consume toxic decaying matter and are in turn prey to fish or birds that further contribute to the cycle of botulism caused by BoNT type E (49, 50). An interesting new dimension to this picture is our finding that, at 10°C, a significant subpopulation of TOX^+^/SPO^+^ cells (31%) activated BoNT expression only during sporulation and exclusively in the forespore. While our system did not allow measurement of BoNT accumulation in spores, earlier reports showed evidence of small amounts of BoNT in spores (51–54) and BoNT/A release from spores upon phagocytosis by leucocytes (55). It is tempting to speculate that in conditions that do not support growth (56–58), minute amounts of BoNT stored in spores and released upon spore germination could suffice to kill or debilitate a host or competitors to generate a growth-supporting environment. This could be particularly advantageous for spores that have been dormant for extended periods and separated from their toxinogenic mother cells or the toxin released upon sporulation (Fig. 6). In contrast, at 30°C, the majority (91%) of sporulating TOX^+^ cells showed BoNT activation in the mother cell compartment but not in the forespore, and BoNT accumulated extracellularly readily upon mother cell lysis. Thus, even during sporulation, the population appears to invest in returning to active growth. This finding is in line with the paradigm that killing a host helps the bacteria to turn their environment into an anaerobic fermenter rich in nutrients, which in turn promotes continued growth (59, 60).

Botulism is a life-threatening paralysis that raises concern among public health professionals and food producers. Botulism can be contracted as an intoxication due to consumption of preformed BoNT with food, feed, or drink or as a toxicoinfection due to spore germination and outgrowth into a toxic culture in the gut or in a deep wound. The low incidence of botulism has been explained by the scarcity of spores in food raw materials (61) and by poor competition of *C. botulinum* in a microbial community (62, 63). We add a new dimension in this discussion by demonstrating that not all actively growing *C. botulinum* cells or cell subpopulations express BoNT in all situations. Further research is needed to understand the environmental and cellular prerequisites for the TOX^−^ states in cells. These factors appear as attractive targets for the development of novel inhibitors of BoNT production.

## SUPPLEMENTAL MATERIAL

Supplemental material mbio.01866-23-s0001.docx Supplemental materials and methods, results, and figures.

ASM does not own the copyrights to Supplemental Material that may be linked to, or accessed through, an article. The authors have granted ASM a non-exclusive, world-wide license to publish the Supplemental Material files. Please contact the corresponding author directly for reuse.
