# Supplementary material for: Cellular and population strategies underpinning neurotoxin production and sporulation in Clostridium botulinum type E cultures
Source: mBio. 2023 Nov 16;14(6):e01866-23. doi: 10.1128/mbio.01866-23 (PMC10746260; doi:10.1128/mbio.01866-23)
Supplement: Supplemental material — s and methods, results, and figures. [file mbio.01866-23-s0001.docx]

# Supplementary Information

**Cellular and population strategies of neurotoxin production and sporulation in *Clostridium botulinum* cultures**

Anna Mertaoja^1^, Gerald Mascher^1^, Maria B. Nowakowska^1^, Hannu Korkeala^1^, Adriano O. Henriques^2^, Miia Lindström^1^*

^1^Department of Food Hygiene and Environmental Health, Faculty of Veterinary Medicine, University of Helsinki, Helsinki, Finland

^2^Instituto de Tecnologia Química e Biológica, Universidade Nova de Lisboa, Lisbon, Portugal

*Corresponding author: Miia Lindström, Department of Food Hygiene and Environmental Health, Faculty of Veterinary Medicine, University of Helsinki, Helsinki, Finland, P. O. Box 66, 00014 University of Helsinki, Finland; phone +358 50 4486587, fax +358 2941 57101, email [miia.lindstrom@helsinki.fi](mailto:miia.lindstrom@helsinki.fi)

# Supplementary materials and methods

**Most probable number and sporulation assays**

To follow growth and sporulation of *Clostridium botulinum* Group II type E strain Beluga, the viable cell and spore counts (MPN/ml or spores/ml, respectively) were measured from 1-ml culture samples. Thirty-microliter aliquots of unheated (total, viable cell counts) or heated (60 °C for 20 min, heat-resistant spore counts) cultures were serially diluted (1:10) in triplicate in fresh TPGY broth on microtiter plates and incubated at 30°C for 48 hours. The plates were visually observed for growth and the most probable numbers of viable cells and spores were calculated according to the Bacteriological Analytical Manual (FDA, <https://www.fda.gov/Food/FoodScienceResearch/LaboratoryMethods/ucm109656.htm>).

**Fluorescence scans and Western blot**

To confirm production of the SNAP^Cd^ in the cell cultures, 1.5-ml culture samples were labeled with 500 nM of TMR-Star for 30 min at 30°C, protected from light, and the cells were washed twice with PBS. The cells were mechanically disrupted in Pathogen Lysis Tubes S (Qiagen, Hilden, Germany) and the total protein concentration in the lysates was determined using the Pierce BCA Protein Assay Kit (Thermo Scientific). Cell lysate samples (10 µg total protein) were mixed 1:1 with 2x sample buffer (2% SDS, 4% glycerol, 0.04 M Tris pH 6.8, 0.01 % bromophenol blue, 0.02 M DTT) and the proteins were resolved in two parallel Any kD™ Mini-PROTEAN® TGX™ Precast Protein Gels (Bio-Rad Laboratories. Inc., Hercules, CA) together with Precision Plus WesternC standard (Bio-Rad). One of the gels was scanned using a Fujifilm FLA-5100 fluorescence scanner, and the other was subjected to detection of the SNAP-tag by immunoblot using an anti-SNAP-tag antibody (New England Biolabs, Ipswich, MA) and a Goat Anti-Rabbit IgG (H+L)-HRP Conjugate (Bio-Rad) antibody at dilutions of 1:1000 and 1:5000, respectively. The immunoblot was scanned using a Fujifilm LAS-3000 imager. Uncropped fluorescence scan and western blot images are available.

# Supplementary results

**Verifying the SNAP-tag signal and single-cell-level analysis of neurotoxin gene expression in *C. botulinum* cells**

To study the botulinum neurotoxin (BoNT) gene expression during growth and sporulation in *C. botulinum* Group II type E strain Beluga, we introduced a reporter plasmid pFT47-P*bontE* containing *SNAP^Cd^* [1] under control of the native BoNT gene promoter region (P*bontE*) (Fig. 1) into our model strain. To optimize the SNAP^Cd^ reporter system for *C. botulinum*, samples of Beluga WT pFT47-P*bontE* and Beluga pFT47 labeled and non-labeled with the fluorescent SNAP-tag substrate TMR-Star were prepared for fluorescent microscopy. As expected, the non-labeled Beluga pFT47-P*bontE* sample showed no fluorescent signal, whereas Beluga pFT47-P*bontE* labeled with 500 nM TMR-Star substrate showed strong fluorescent signal as a sign of P*bontE* activation and subsequent successful SNAP^Cd^ labeling (Fig. S1a). Beluga pFT47 cells labeled with TMR-Star substrate showed a weak background fluorescent signal (Fig. S1a). Thus, in the subsequent experiments, Beluga pFT47 samples labeled with TMR-Star substrate were used as a negative background control and the signal obtained for images of the Beluga pFT47 cultures was subtracted from the signal obtained for images of the strain Beluga pFT47-P*bontE*.

To further ensure that the fluorescent signal was caused by SNAP^Cd^ production and not by autofluorescence or unspecific binding of the TMR-Star substrate, SDS-PAGE and fluorescent scans were performed. In the fluorescent scans, fluorescent signal was only detected in the Beluga pFT47-P*bontE* sample labeled with TMR-Star substrate, indicating the lack of autofluorescence or unspecific substrate binding (Fig. S1b). Western blot analysis using the anti-SNAP-tag antibody showed that all of the SNAP^Cd^ produced by Beluga pFT47-P*bontE* was labeled, which allows the use of the SNAP^Cd^ as a quantitative as well as qualitative reporter, and that the Beluga pFT47 negative control strain did not produce the SNAP^Cd^ reporter protein (Fig. S1c).

P*bontE* activity was first studied in our routine growth conditions (30 °C, TPGY broth) in Beluga wild-type strain in three independent replicates. Growth was followed by optical density measurements. Samples were collected for phase-contrast and fluorescence microscopy and neurotoxin ELISA assays at 6, 9, 11, 14, 17, 20, 24, 48 and 96 hours after inoculation. Fluorescence microscopy samples were labeled with TMR-Star and membrane dye Mitotracker Green (MTG) to monitor P*bontE-SNAP^Cd^* activation and to visualize cell membranes, respectively. Heat-resistant spore counts were studied at 96 hours after inoculation.

P*bontE-SNAP^Cd^* production (further referred to as TOX+ cells) was detected already at early logarithmic growth phase in a small sub-population of cells (10‒20 % of population depending on the replicate) in the wild-type strain (Figure S2a and b, and Fig. S3). The percentage of TOX+ cells peaked (80‒90 % of population) at transition from logarithmic to stationary phase, and declined rapidly in early stationary phase. In late-stationary cultures, less than 2 % of cells showed neurotoxin promoter activity. The results are in agreement with earlier reports [2–4] showing neurotoxin gene expression patterns to peak at late-logarithmic to early stationary phase. However, the time window showing TOX+ cells in fluorescence microscopy was generally wider than with mRNA-based assays [4] and showed that neurotoxin gene synthesis may be initiated in response to early-log-phase events. This also demonstrates that the single-cell-level fluorescence-based neurotoxin gene expression analysis provides a more sensitive overview on toxin gene expression in *C. botulinum* cultures than conventional mRNA-based assays.

The culture grown in TPGY at 30 °C was dominated by cells showing no division septum (80‒100 % depending on the sampling time point), suggesting slow growth and thus a relatively long generation time. Only in the mid-log-phase cultures where growth is expected to be fastest, approximately 14‒20 % of cells were dividing symmetrically, whereas in late-log and stationary phases only 0‒5 % of the cells were dividing. Both TOX+ and TOX- cells were detected among the non-dividing, symmetrically dividing, and asymmetrically dividing cell populations, suggesting that BoNT production is unrelated to cell division or commitment to sporulation. Sporulating cells or mature spores were not microscopically detectable in the cultures grown in TPGY, but spore assays revealed a relatively low concentration of heat-resistant spores (approx. 10^4^-10^5^ spores/ml, total cell count 10^8^ MPN/ml). Considering the low sporulation frequency, the massive cell lysis and resulting large numbers of decaying cells and cell debris detected during stationary phase were likely not the result of mother cell lysis upon spore release, but autolysis of vegetative cells.

**Cultures started from a single colony have differently behaving subpopulations**

To explore the effect of a homogeneous inoculum on the population structure, we inoculated xx-ml aliquots of fresh CMM-TPGY with single colonies of Beluga pFT47-P*bontE* picked from a TPGY agar plate. Fluorescence microscopy revealed that despite using an inoculum that is generally considered to originate from a single cell, the populations showed heterogeneity and differently behaving subpopulations (Fig. S5). We thus conclude that heterogeneity, at least to some degree, is an inherent trait in *C. botulinum* Beluga cultures. The data may suggest that the inoculum has an effect on the proportions of different subpopulations.

**Toxin promoter activation accurately reports on toxin production**

The single-cell-level fluorescence-based neurotoxin gene promoter analysis was in agreement with neurotoxin ELISA measurements, showing accumulation of intracellular BoNT soon after detection of the first TOX+ cell population in TPGY broth (Figure S2c). Intracellular BoNT levels declined accordingly with a decrease in the TOX+ cell population. The accumulation of BoNT in the culture supernatant correlated strongly (Pearson’s *r* = -0.83, p=0.001) with the declining optical density of the culture at transition phase. By the end of the experiment a total BoNT concentration of 2748 ng/ml was reached. Fourteen % of BoNT remained intracellular and 86 % was released in the culture supernatant. Heat-resistant spore count assays and phase-contrast microscopy suggested the decline in optical density to be primarily due to cell lysis, and to a minor extent due to sporulation. Accordingly, relatively low spore counts of 10^3^‒10^4^ spores/ml were detected in the wild-type cultures grown in TPGY. Moreover, less than 2 % of cells showed asymmetric cell division and no spores nor forespores were visible by microscopy in any of the samples examined.

In bi-phasic CMM-TPGY medium positive correlation (Pearson’s *r* = 0.69, p=0.001) was detected between the percentage of TOX+ cells in the population and the intracellular neurotoxin concentration at the same sampling time point. The total neurotoxin concentration reached 2890 ng/ml, with only 3% of the toxin remaining in the cells and 97% being released to the culture supernatant (Figure 2c). A strong negative correlation (Pearson’s *r* = -0.96, p=0.002) was discovered between the accumulation of BoNT in the culture supernatant and the decline in optical density. Low levels of BoNT were detected in the culture supernatant already during logarithmic growth (Figs. 2 and S4). Due to decline in the most probable number of the cultures between 12 h (1.4x10^8^-1.4x10^9^ MPN/ml) and 18 h (8.0x10^7^-2.5x10^8^ MPN/ml), the accumulation of BoNT in the culture supernatant is likely the result of cell lysis, either autolysis or mother cell lysis upon sporulation. The latter is supported by a low number of spores observed already at 12 and 18 h (10^2^-10^4^ spores/ml). Due to the high spore concentration (10^7^‒10^8^ spores/ml) at the stationary growth phase, toxin release in these conditions was assumed to be primarily due to mother cell lysis upon sporulation.

The findings described above provide a reliable basis for single-cell-level fluorescence-based neurotoxin gene expression analysis in *C. botulinum.* Importantly, not all cells contribute to the net level of BoNT produced in the culture. This demonstrates heterogeneity in toxin production within a population.

***spo0A* complementation successfully restores BoNT production and sporulation**

To ensure that the changes in phenotype observed in the Δ*spo0A*::bm pFT47-P*botE* cultures were caused by *spo0A* deletion and not by polar effects, we introduced the pFT47-P*botE* plasmid into complemented strain Beluga Δ*spo0A*::bm::*spo0A*-wm [5]. Fluorescence microscopy and neurotoxin ELISA as well as spore heating assays showed WT-level presence of TOX+ cells in the population and successful restoration of neurotoxin production and sporulation (Figure S8).

**References**

[1] F.C. Pereira, L. Saujet, A.R. Tomé, M. Serrano, M. Monot, E. Couture-Tosi, I. Martin-Verstraete, B. Dupuy, A.O. Henriques, The Spore Differentiation Pathway in the Enteric Pathogen Clostridium difficile, PLoS Genet. (2013). https://doi.org/10.1371/journal.pgen.1003782.

[2] M. Bradshaw, S.S. Dineen, N.D. Maks, E.A. Johnson, Regulation of neurotoxin complex expression in Clostridium botulinum strains 62A, Hall A-hyper, and NCTC 2916, Anaerobe. (2004). https://doi.org/10.1016/j.anaerobe.2004.07.001.

[3] I. Artin, A.T. Carter, E. Holst, M. Lövenklev, D.R. Mason, M.W. Peck, P. Rådström, Effects of carbon dioxide on neurotoxin gene expression in nonproteolytic Clostridium botulinum type E, Appl. Environ. Microbiol. (2008). https://doi.org/10.1128/AEM.02587-07.

[4] Y. Chen, H. Korkeala, J. Lindén, M. Lindström, Quantitative real-time reverse transcription-PCR analysis reveals stable and prolonged neurotoxin cluster gene activity in a clostridium botulinum type E strain at refrigeration temperature, Appl. Environ. Microbiol. (2008). https://doi.org/10.1128/AEM.00469-08.

[5] A. Mertaoja, M.B. Nowakowska, G. Mascher, V. Heljanko, D. Groothuis, N.P. Minton, M. Lindström, CRISPR-Cas9-based toolkit for Clostridium botulinum Group II spore and sporulation research, Front. Microbiol. 12 (2021) 32. https://doi.org/10.3389/FMICB.2021.617269.

# Supplementary figures


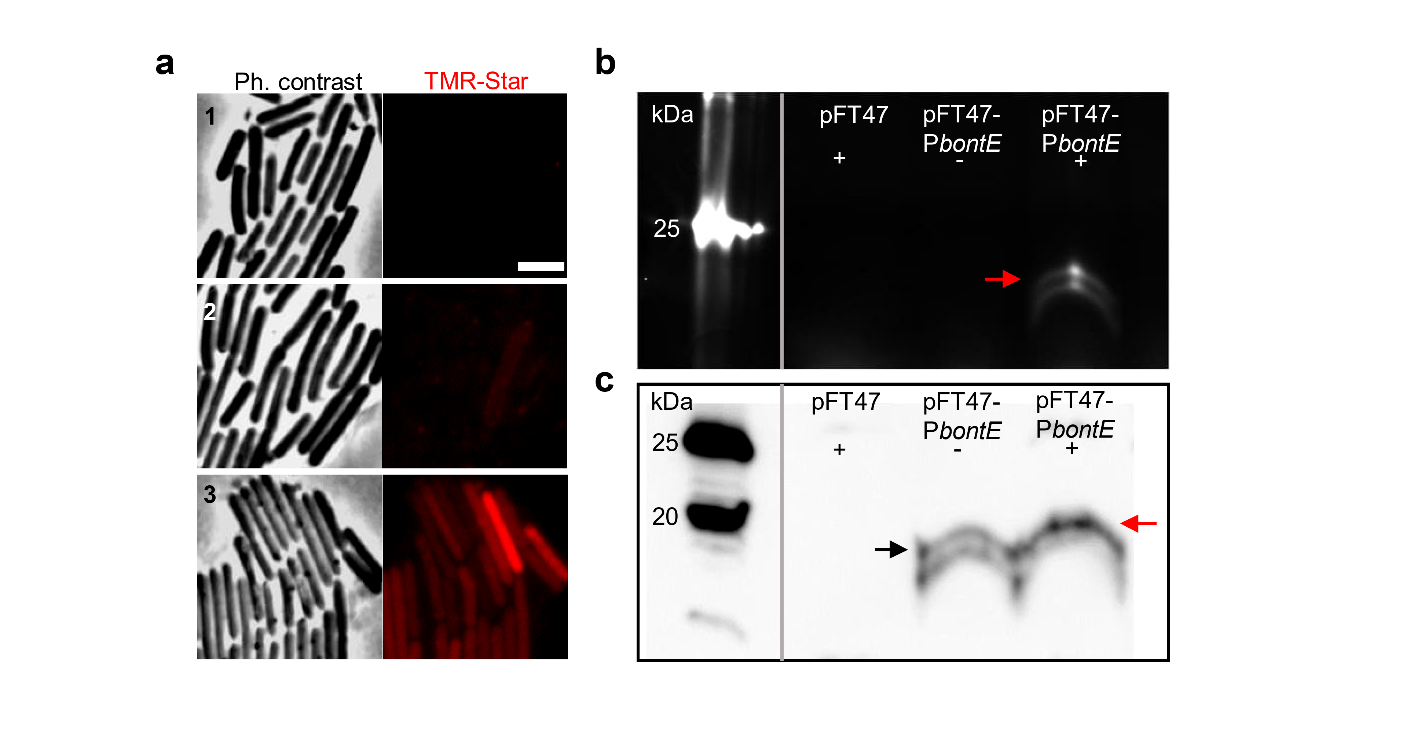


**Figure S1. Verification of the SNAP-tag reporter system in *Clostridium botulinum* strain Beluga**. (**a**) Fluoresence signal of non-labeled Beluga pFT47-P*bontE* (**1**), Beluga pFT47 labeled with TMR-Star substrate (**2**), and Beluga pFT47-P*bontE* labeled with TMR-Star substrate (**3**). Cultures were grown until late logarithmic growth phase and samples (**2**) and (**3**) were labeled with 500 nM TMR-Star substrate before fluorescence microscopy. Non-labeled Beluga pFT47-P*bontE* (**1**) did not show any fluorescent signal. Labeled negative control Beluga pFT47 (**2**) showed a weak background signal. Beluga pFT47-P*bontE* (**3**) labeled with TMR-Star substrate showed a strong fluorescent signal in the majority of the cells, indicating the accumulation of SNAP^Cd^ reporter protein in the cells. (**b**) and (**c**) Whole-cell samples of Beluga pFT47-P*bontE* and Beluga pFT47 were labeled with TMR-Star (indicated by “+”, non-labeled controls “-“) and the proteins were resolved in two parallel SDS-PAGE gels after disrupting the cells. The first gel was scanned with a fluorescent scanner (**b**), and the other subjected to immunoblot with an anti-SNAP-tag antibody (**c**). Red and black arrows point to labeled and non-labeled SNAP^Cd^ protein, respectively. Grey lines indicate the removal of empty lanes between standard and pFT47 sample. Size bar 5 $\mu$M.


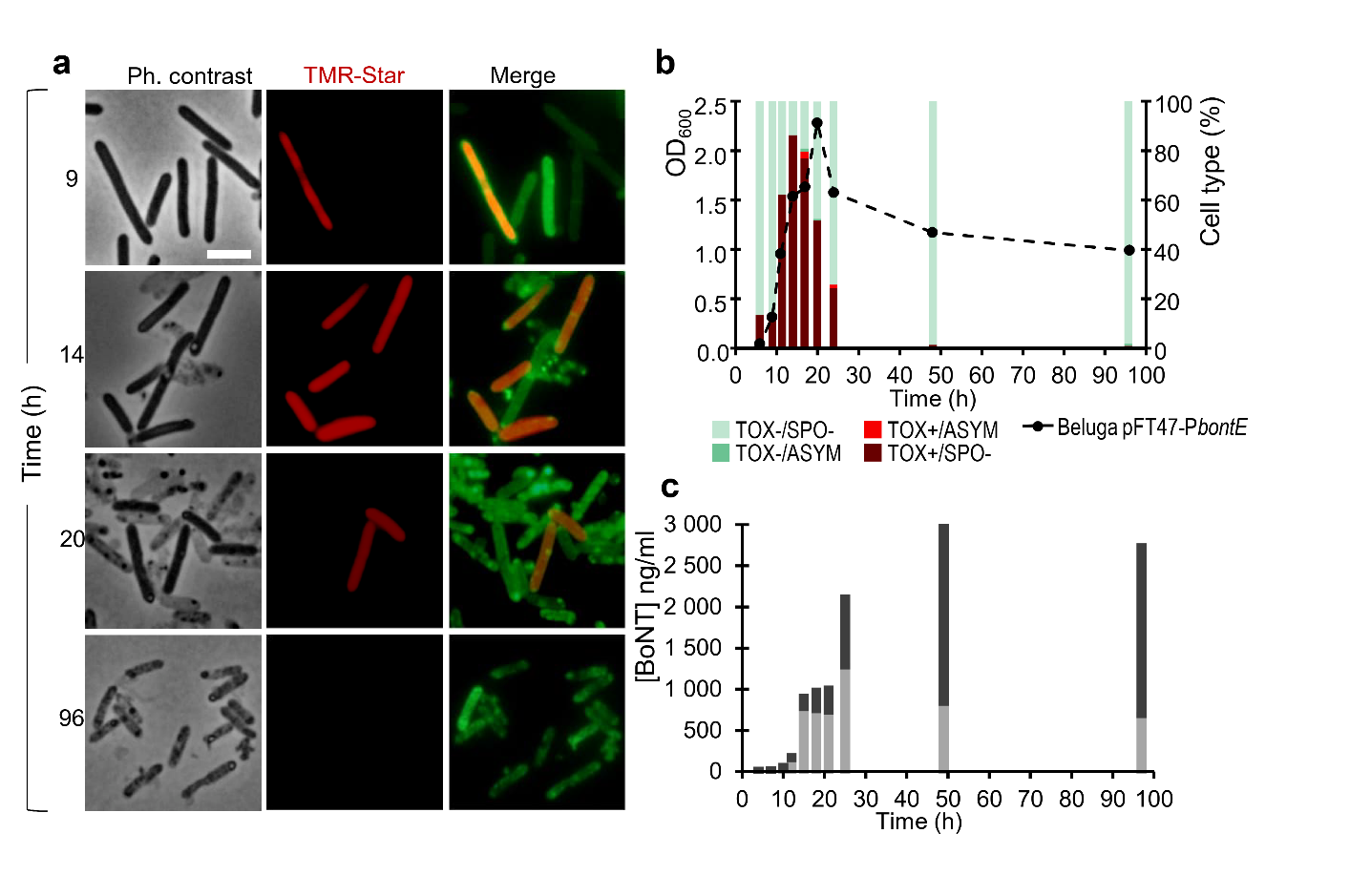


**Figure S2.** **Growth, neurotoxin gene promoter activation, and botulinum neurotoxin production of *Clostridium botulinum* strain Beluga pFT47-P*bontE* in TPGY broth.** (**a**) To determine the neurotoxin gene promoter activation, samples were collected and stained with TMR-Star substrate at indicated time points after inoculation. Membrane dye MTG (green) was used to visualize cell membranes. The samples were examined by phase-contrast and fluorescent (TMR-Star and MTG) microscopy to detect dividing or sporulating cells and cells producing SNAP^Cd^ as an indicator of toxin gene promoter activation (TOX+). The merged images show the overlap between TMR-Star (red) and MTG (green) channels. Lighter grey cells with irregular shape visible in phase-contrast images at later time points were considered non-viable and excluded from analysis. (**b**) Population structure at each sampling time-point concerning toxin promoter activation (TOX+ or TOX-) and sporulation (SPO- = vegetative cell; ASYM = asymmetrically dividing cell). The total number of cells scored for each time point were 9 hours, n = 295; 11 hours, n = 37; 14 hours, n = 290; 17 hours, n = 224; 20 hours, n = 261; 24 hours, n = 74; 48 hours, n = 221: 96 hours, n = 219. Growth was followed by measuring optical density at 600 nm. (**c**) Neurotoxin concentration in cell sediments (light grey bars) and culture supernatants (dark grey bars) was determined by ELISA. Data shown are from one representative experiment out of three independent experiments. See Fig. S3 for other replicates. Size bar 5 $\mu$M.


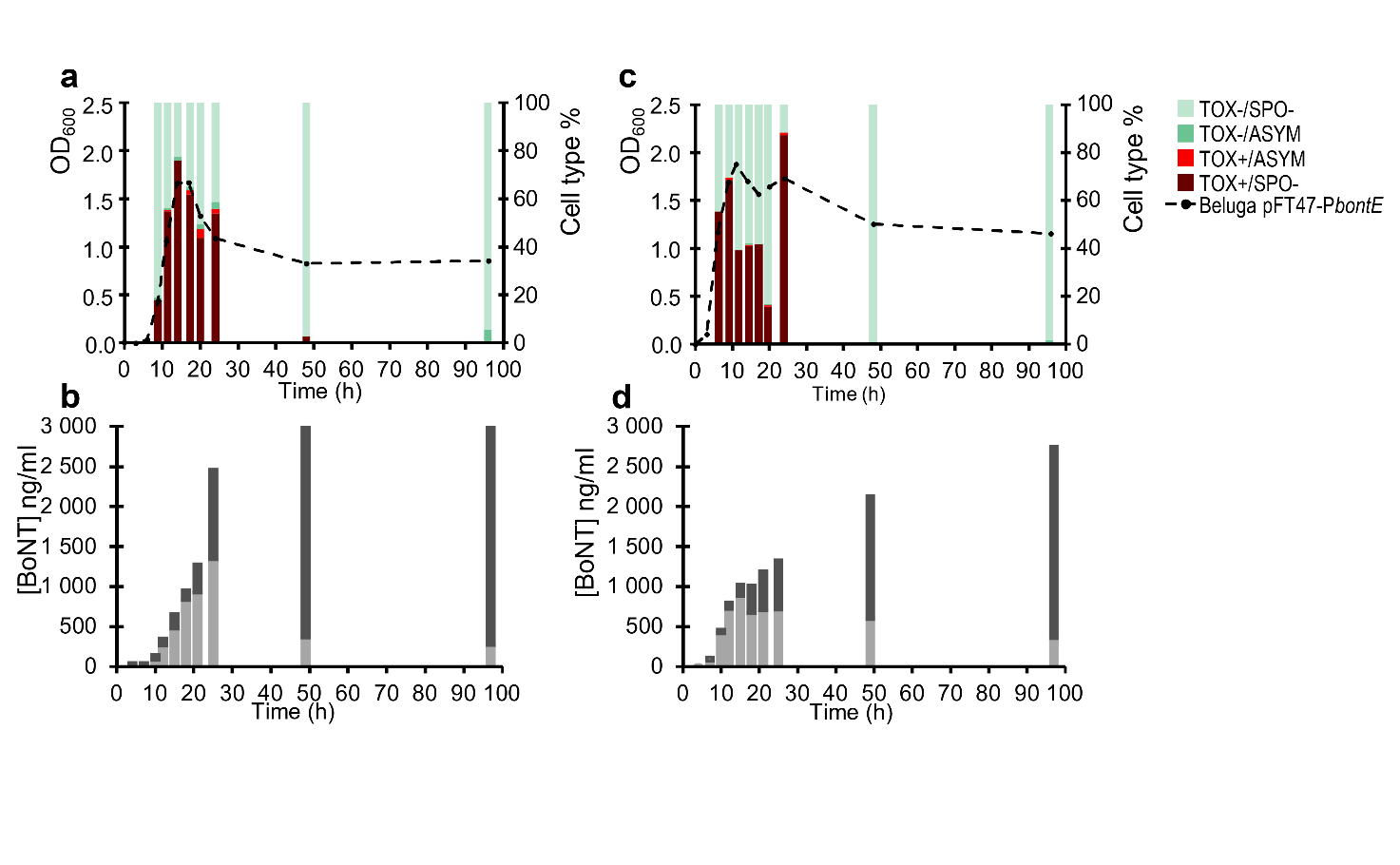


**Figure S3**. **Growth, neurotoxin gene promoter activation, and botulinum neurotoxin production of *C. botulinum* E1 Beluga pFT47-P*bontE* in TPGY broth, replicates.** (**a** and **c**) Population structure of Beluga pFT47-P*bontE* concerning toxin promoter activation (TOX+ or TOX-) and sporulation (SPO- = vegetative cell; ASYM = asymmetrically dividing cell). Growth was followed by measuring optical density at 600 nm. The total numbers of cells scored at each sampling time point were for (**a**) 9 hours, n = 90; 11 hours, n = 170; 14 hours, n = 149; 17 hours, n = 159; 20 hours, n = 55; 24 hours, n = 324; 48 hours, n = 83: 96 hours, n = 171; and (**c**) 6 hours, n = 96; 9 hours, n = 72; 11 hours, n = 120; 14 hours, n = 208; 17 hours, n = 130; 20 hours, n = 186; 24 hours, n = 92; 48 hours, n = 125: 96 hours, n = 123. (**b** and **d**) Neurotoxin concentration in cell sediments (light grey bars) and culture supernatants (dark grey bars) was determined by ELISA.


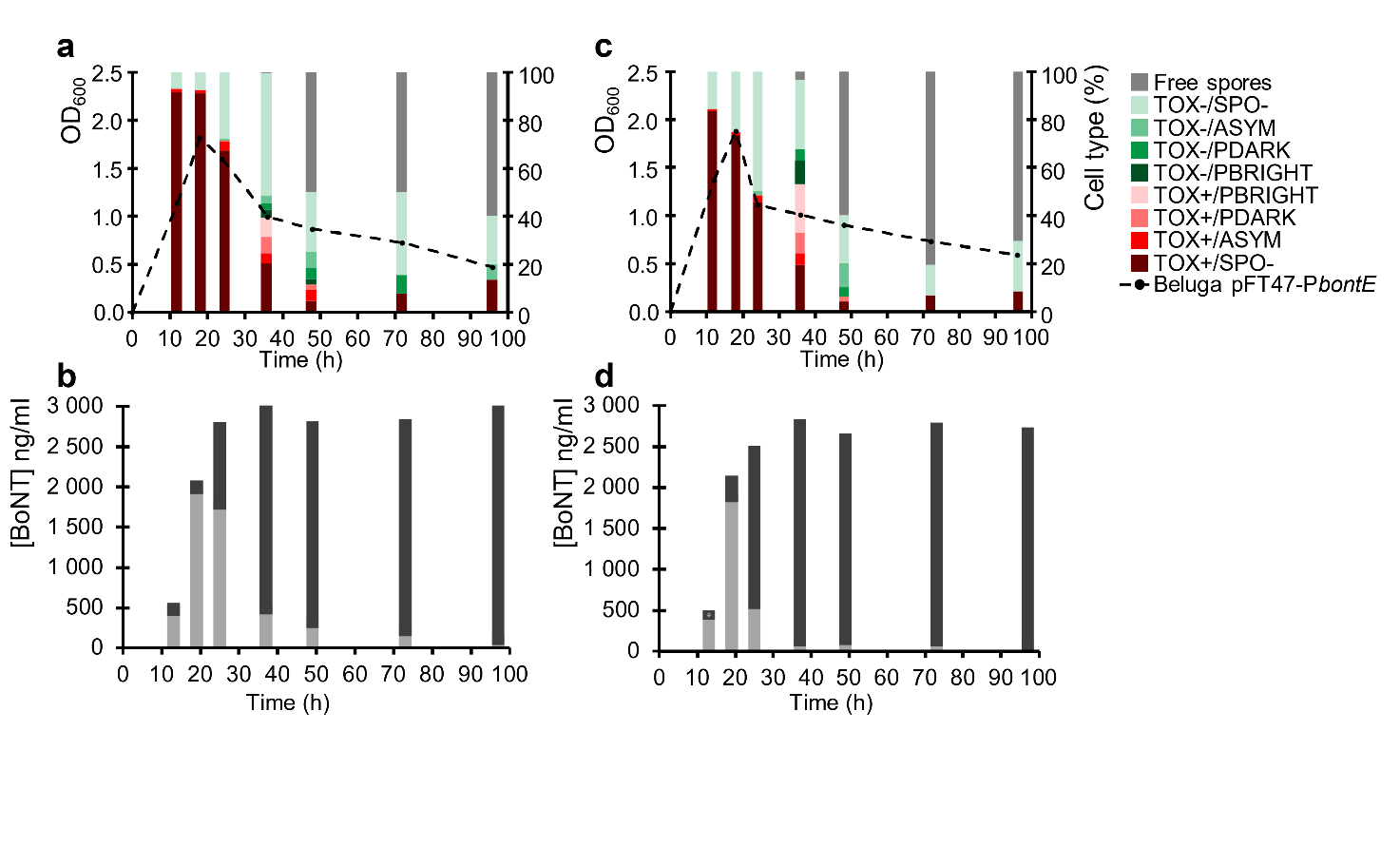


**Figure S4. Growth, neurotoxin gene promoter activation, and botulinum neurotoxin production of *C. botulinum* E1 Beluga pFT47-P*bontE* in CMM-TPGY at 30 °C, replicates.** (**a** and **c**) Population structure of Beluga pFT47-P*bontE* concerning toxin promoter activation (TOX+ or TOX-) and sporulation (SPO- = vegetative cell; ASYM = asymmetrically dividing cell; PDARK = phase-dark forespore; PBRIGHT = phase bright forespore). Growth was followed by measuring optical density at 600 nm. The total number of cells scored for each time point for (**a**) were at 12 hours, n = 684; 18 hours, n = 1063; 24 hours, n = 458; 36 hours, n = 285; 48 hours, n = 44; 72 hours, n = 26; 96 hours, n = 15, and for (**c**) at 12 hours, n = 633; 18 hours, n = 685; 24 hours, n = 435; 36 hours, n = 83; 48 hours, n = 50; 72 hours, n = 31; 96 hours, n = 24. (**b** and **d**) Neurotoxin concentration in cell sediments (light grey bars) and culture supernatants (dark grey bars) was determined by ELISA.


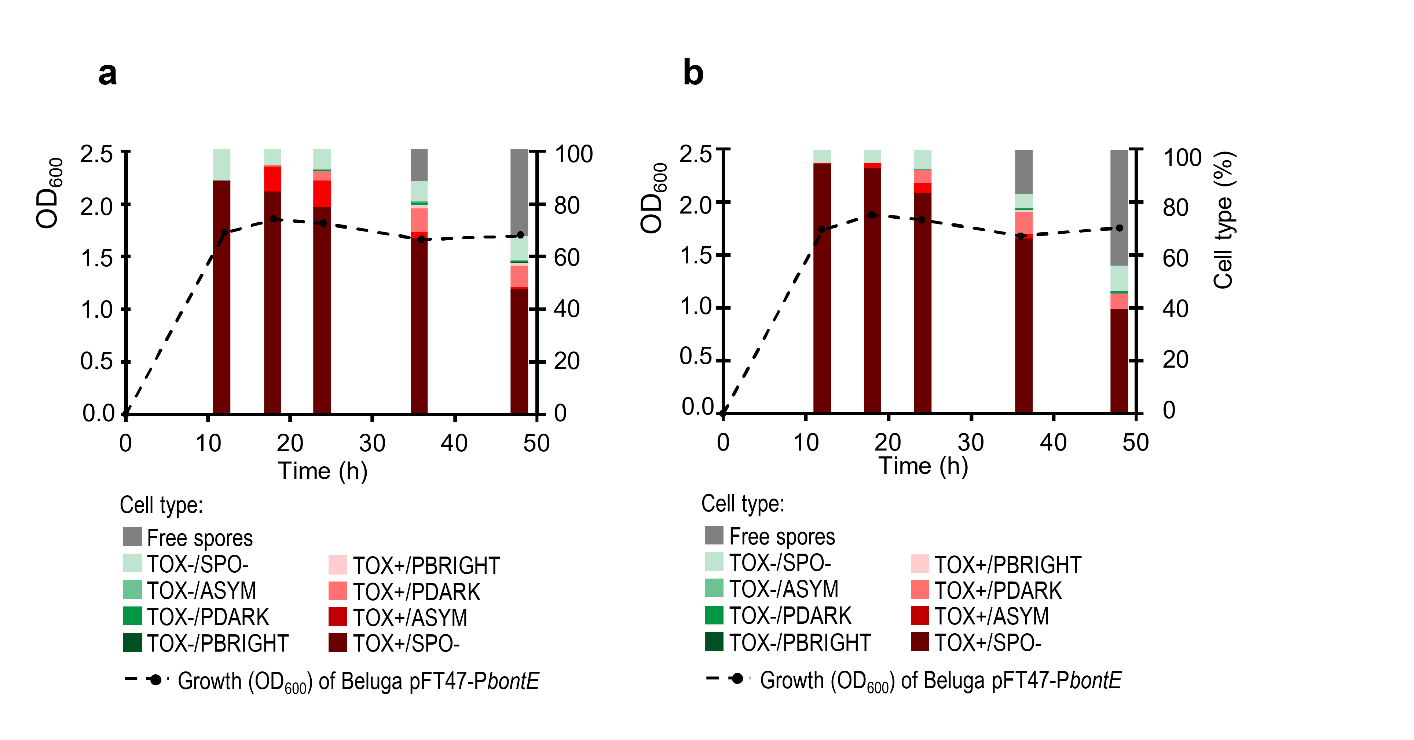


**Figure S5. Growth and neurotoxin gene promoter activation of *C. botulinum* E1 Beluga pFT47-P*bontE* cultures started from a single colony in CMM-TPGY at 30 °C.** (**a** and **b**) Population structure of Beluga pFT47-P*bontE* concerning toxin promoter activation (TOX+ or TOX-) and sporulation (SPO- = vegetative cell; ASYM = asymmetrically dividing cell; PDARK = phase-dark forespore; PBRIGHT = phase bright forespore). A single colony from an agar plate was used as inoculum to test whether homogeneous inocula result in heterogeneous populations. Growth was followed by measuring optical density at 600 nm. The total number of cells scored for each time point for (**a**) were at 12 hours, n = 495; 18 hours, n = 723; 24 hours, n = 577; 36 hours, n = 490; 48 hours, n = 569; and for(**b**) at 12 hours, n = 548; 18 hours, n = 672; 24 hours, n = 532; 36 hours, n = 591; 48 hours, n = 502.


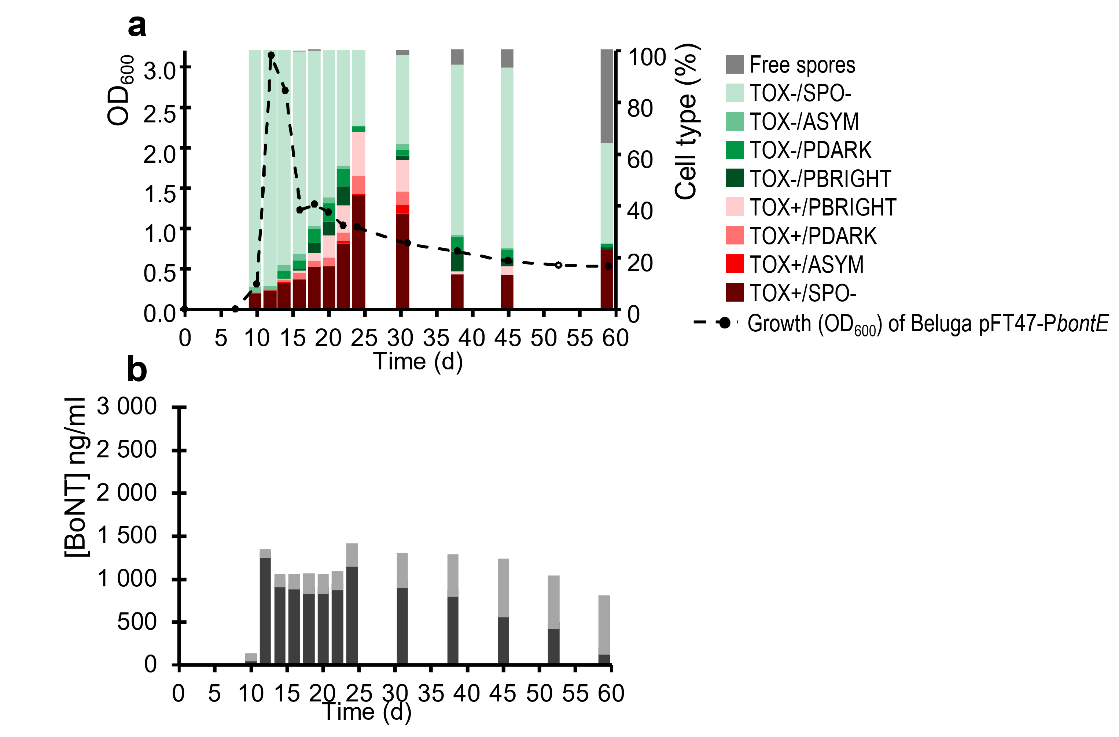


**Figure S6. Growth, neurotoxin gene promoter activation, and botulinum neurotoxin production of *C. botulinum* E1 Beluga pFT47-P*bontE* in CMM-TPGY at 10 °C, replicates.** (**a**) Population structure of Beluga pFT47-P*bontE* concerning toxin promoter activation (TOX+ or TOX-) and sporulation (SPO- = vegetative cell; ASYM = asymmetrically dividing cell; PDARK = phase-dark forespore; PBRIGHT = phase bright forespore). The total number of cells scored for each time point were 10 days, n = 498; 12 days, n = 1125; 14 days, n = 1182; 16 days, n = 920; 18 days, n = 787; 20 days, n = 645; 22 days, n = 551; 24 days, n = 438; 31 days, n = 179; 38 days, n = 105; 45 days, n = 215; 60 days, n = 80. Growth was followed by measuring optical density at 600 nm. (**b**) Neurotoxin concentration in cell sediments (light grey bars) and culture supernatants (dark grey bars) was determined by ELISA.


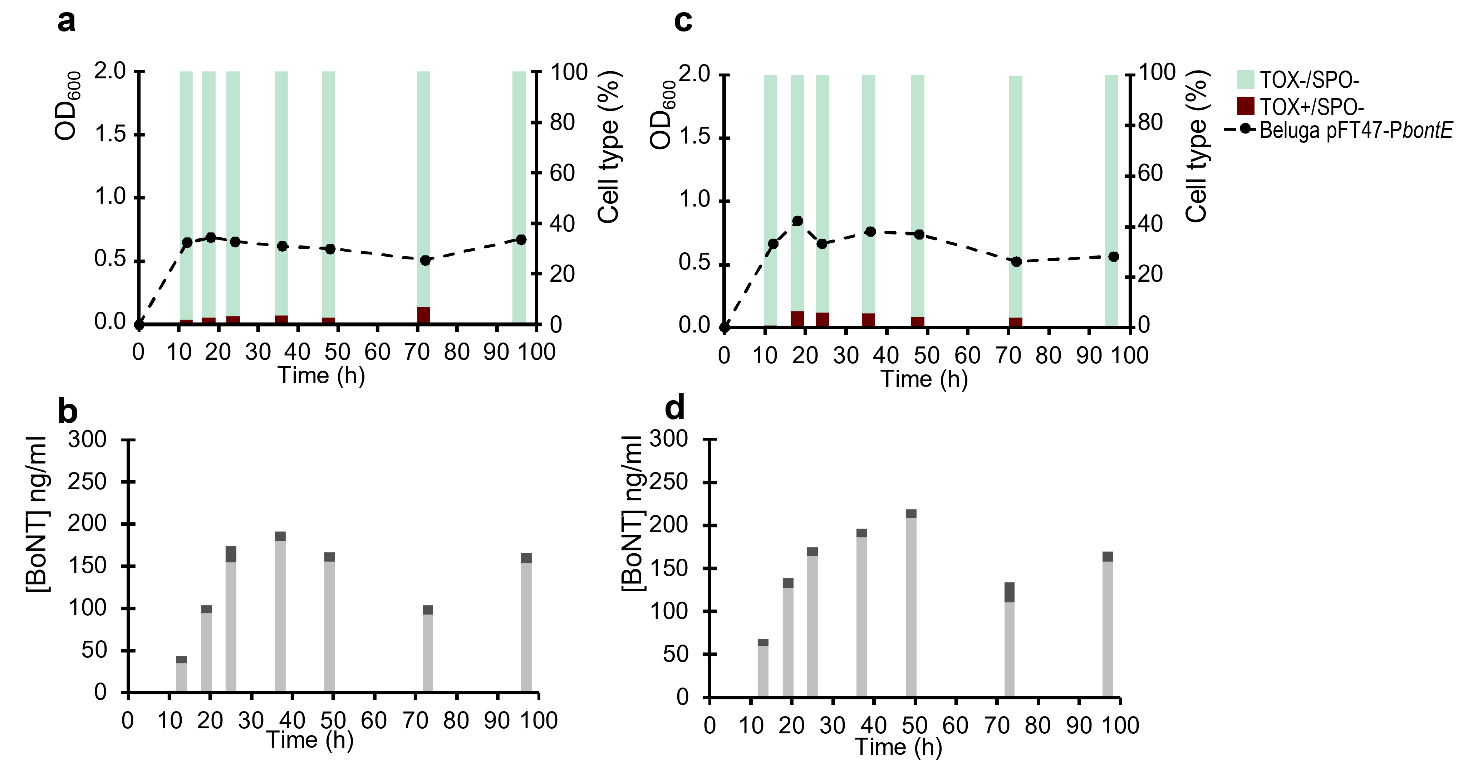


**Figure S7. Growth, toxin gene promoter activation, and botulinum neurotoxin production of Beluga Δ*spo0A* pFT47-P*bontE* in CMM-TPGY at 30 °C, replicates.** (**a** and **c**) Population structure of Beluga pFT47-P*bontE* concerning toxin promoter activation (TOX+ or TOX-) and sporulation. All observed cells were non-sporulating vegetative cells (SPO-). The total number of cells scored for each time point were for **a,** 12 hours, n = 1107; 18 hours, n = 346; 24 hours, n = 2422; 36 hours, n = 640; 48 hours, n = 608; 72 hours, n = 424; 96 hours, n = 703, and for **c,** 12 hours, n = 893; 18 hours, n = 584; 24 hours, n = 742; 36 hours, n = 689; 48 hours, n = 884; 72 hours, n = 679; 96 hours, n = 864. Growth was followed by measuring optical density at 600 nm. (**b** and **d**) Neurotoxin concentration in cell sediments (light grey bars) and culture supernatants (dark grey bars) was determined by ELISA.


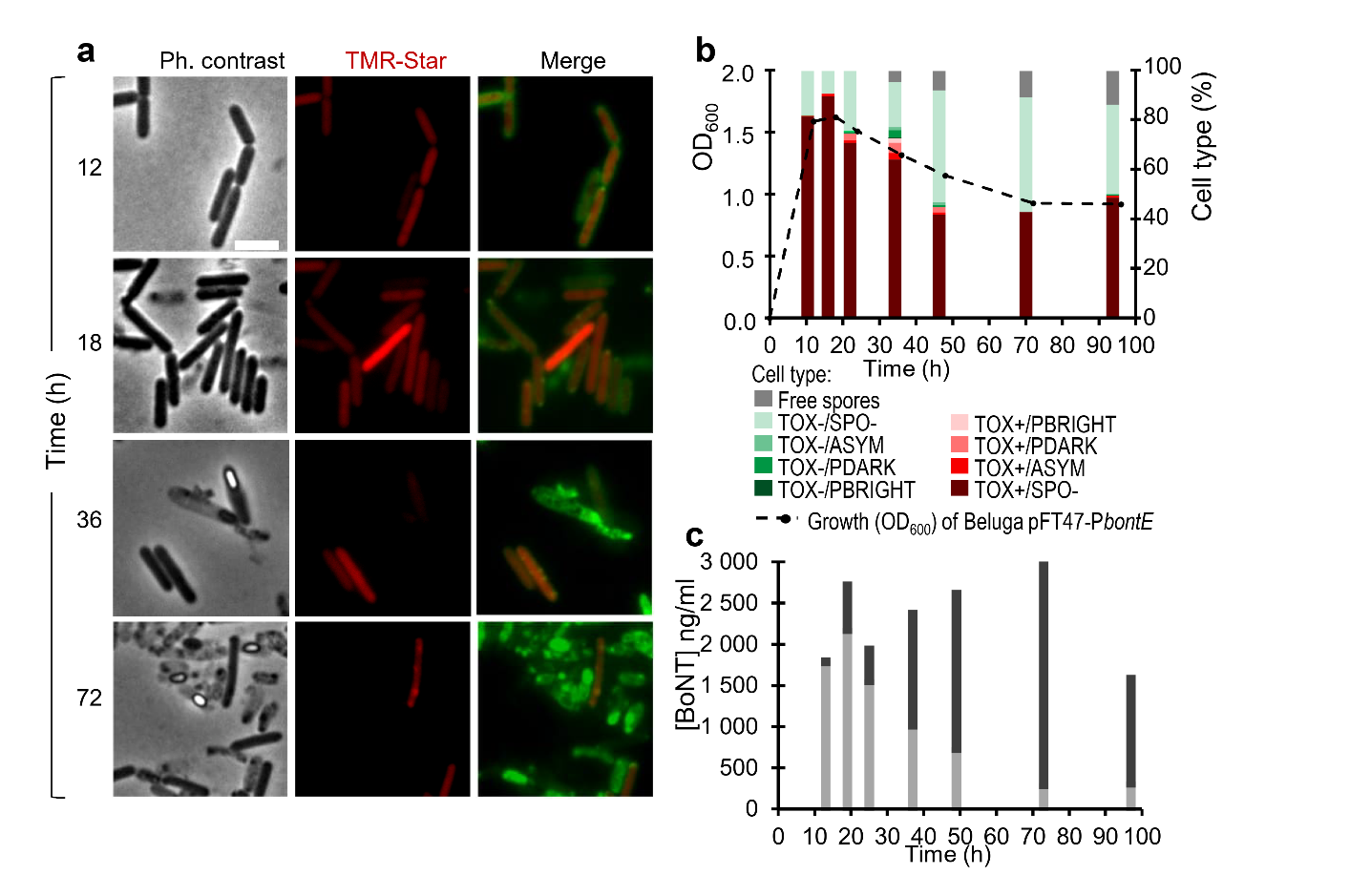


**Figure S8. Growth, toxin gene promoter activation, and botulinum neurotoxin production of Beluga *Δspo0A::spo0A* pFT47-P*bontE* in CMM-TPGY at 30 °C.** (**a**) To determine the toxin gene promoter activation, samples were collected and labeled with TMR-Star substrate and membrane dye MTG at indicated time points after inoculation and examined by phase-contrast (PC) and fluorescent (TMR-Star and MTG) microscopy to monitor sporulation and SNAP^Cd^ production. The merged images show the overlap between TMR-Star (red) and MTG (green) channels. (**b**) Population structure at each sampling time-point concerning toxin production (TOX+ or TOX-) and sporulation (SPO- = vegetative cell; ASYM = asymmetrically dividing cell; PDARK = phase-dark forespore; PBRIGHT = phase bright forespore) The total number of cells scored for each time point were 12 hours, n = 226; 18 hours, n = 398; 24 hours, n = 344; 36 hours, n = 239; 48 hours, n = 224; 72 hours, n = 229; 96 hours, n = 167. Growth was followed by measuring optical density at 600 nm. (**c**) Neurotoxin concentration in cell pellets (light grey bars) and culture supernatants (dark grey bars) were determined with ELISA. Data shown are from one representative experiment out of three independent experiments. Size bar 5 $\mu$M.
